# Supplementary material for: Selenium-based metabolic oligosaccharide engineering strategy for quantitative glycan detection
Source: Nat Commun. 2023 Dec 13;14:8281. doi: 10.1038/s41467-023-44118-w (PMC10719347; doi:10.1038/s41467-023-44118-w)
Supplement: Supplementary file 1 — Supplementary Information [file 41467_2023_44118_MOESM1_ESM.pdf]

SUPPLEMENTARY INFORMATION FILE

Selenium-based metabolic oligosaccharide engineering strategy for quantitative glycan detection

This Supplementary Information includes **Supplementary Figs. 1-44**, **Supplementary Notes 1-4**, **Supplementary Tables 1-5**:

Supplementary Fig. 1: Cytotoxicity of SeMOE probes
Supplementary Fig. 2: Sialic acid levels of SeMOE-treated cells
Supplementary Fig. 3: Selenoprotein mRNA levels of SeMOE-treated cells
Supplementary Fig. 4: Calibration curve of Se standard solution
Supplementary Fig. 5: Se levels of glycoproteins from SeMOE-treated HeLa cells
Supplementary Fig. 6: CyTOF analysis of SeMOE-treated MCF-7 cells
Supplementary Fig. 7: Linear correlation between cell counts and Se level
Supplementary Fig. 8: S-glyco-modification of SeMOE probes
Supplementary Fig. 9: Se levels of glycoproteins from respective SeMOE probe-labelled HeLa cells treated with PNGase F or P-3F_{AX}-Neu5Ac
Supplementary Fig. 10: Metabolic competition analysis of SeMOE
Supplementary Fig. 11: DMB-derived sialic acid analysis of SeMOE-treated HeLa cells
Supplementary Fig. 12: SeMOE of 1,6-Pr_2GalNSe via GalNAc salvage pathway
Supplementary Fig. 13: Metabolic labeling of HeLa cells by 9AzSiaNSe
Supplementary Fig. 14: Se level of different subcellular proteins in MCF-7 cells
Supplementary Fig. 15: Examples of glycopeptides in pGlyco3 analysis
Supplementary Fig. 16: Comparison of the molecular weight of glycopeptides identified in pGlyco3 and SESTAR++ searches
Supplementary Fig. 17: Applicability of SeMOE in Jurkat and K562 cells
Supplementary Fig. 18: Glycan transfer during Jurkat cell-K562 cell trogocytosis
Supplementary Fig. 19: Systematic evaluation of SeMOE in various cancer cell lines
Supplementary Fig. 20: Seleno-sialic acid transfer assay during 4T1 and RAW 264.7 cells co-incubation
Supplementary Fig. 21: Seleno-glycan transfer during cancer cell and RAW 264.7 cell co-incubation
Supplementary Fig. 22: Biological effects of co-incubation of cancer cells and RAW 264.7 cells
Supplementary Fig. 23: Imaging of sialoglycoconjugates after GC-oocyte interaction
Supplementary Fig. 24: Body weight of SeMOE-treated BALB/c mice

Supplementary Fig. 25: Selenoamino acid levels of SeMOE-treated mice
Supplementary Fig. 26: *In situ* visualization of sialoglycans in mouse tissues
Supplementary Fig. 27: ROS assay of various cell lines after SeMOE treatment
Supplementary Fig. 28: ROS assay of respective monosaccharide-treated cells
Supplementary Fig. 29: Cell migration assay of MCF-7 cells after SeMOE treatment
Supplementary Fig. 30: Apoptosis assay of MCF-7 cells after SeMOE treatment
Supplementary Fig. 31: ^{1}H NMR and ^{13}C NMR spectrum of **3**
Supplementary Fig. 32: ^{1}H NMR and ^{13}C NMR spectrum of **4**
Supplementary Fig. 33: ^{1}H NMR and ^{13}C NMR spectrum of **7a**
Supplementary Fig. 34: ^{1}H NMR and ^{13}C NMR spectrum of **7b**
Supplementary Fig. 35: ^{1}H NMR and ^{13}C NMR spectrum of **8a**
Supplementary Fig. 36: ^{1}H NMR and ^{13}C NMR spectrum of **8b**
Supplementary Fig. 37: ^{1}H NMR and ^{13}C NMR spectrum of **13a**
Supplementary Fig. 38: ^{1}H-^{1}H COSY NMR and ^{1}H-^{13}C HSQC NMR spectrum of **13a**
Supplementary Fig. 39: ^{1}H NMR and ^{13}C NMR spectrum of **13b**
Supplementary Fig. 40: ^{1}H-^{1}H COSY NMR and ^{1}H-^{13}C HSQC NMR spectrum of **13b**
Supplementary Fig. 41: ^{1}H NMR and ^{13}C NMR spectrum of **14**
Supplementary Fig. 42: ^{1}H NMR and ^{13}C NMR spectrum of **15**
Supplementary Fig. 43: ^{1}H NMR and ^{13}C NMR spectrum of **16**
Supplementary Fig. 44: ^{1}H NMR and ^{13}C NMR spectrum of **17**

Supplementary Note 1: Synthetic procedures
Supplementary Note 2: CyTOF gating strategy
Supplementary Note 3: Gating strategy in Jurkar-K562 trogocytosis experiment
Supplementary Note 4: Calculation of the number of selenoproteins in a human body

Supplementary Table 1: Instrumentation and measurement parameters of ICP-MS in solution
Supplementary Table 2: Instrumental parameters of LA-ICP-MS for selenoprotein analysis on PVDF membrane
Supplementary Table 3: Instrumental parameters of LA-ICP-TOF MS for mouse tissue sialoglycan imaging
Supplementary Table 4: Instrumental parameters of LA-ICP-MS for mouse tissue sialoglycan quantification *in situ*
Supplementary Table 5: RT-qPCR primers

Supplementary Figures

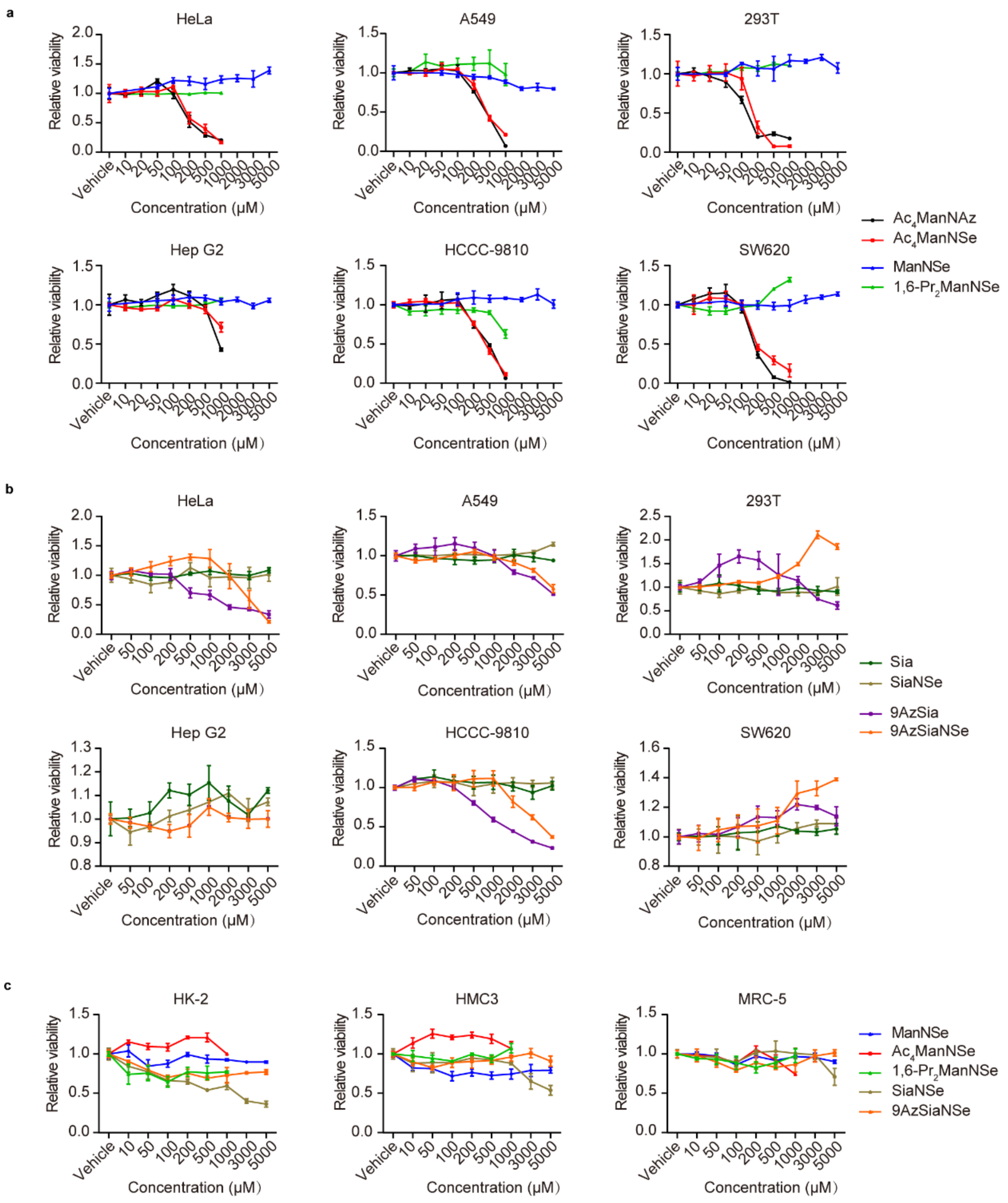

Supplementary Fig. 1 Cytotoxicity of SeMOE probes. A variety of cells were incubated with various SeMOE probes, in a concentration ranging from 0-5 mM (as designated in each chart), for 48 h. The cells were then analyzed using commercialized CCK-8 cell viability assay. Error bars represent mean ± SD of n = 4 independent biological replicates. Source data are provided as a Source Data file.

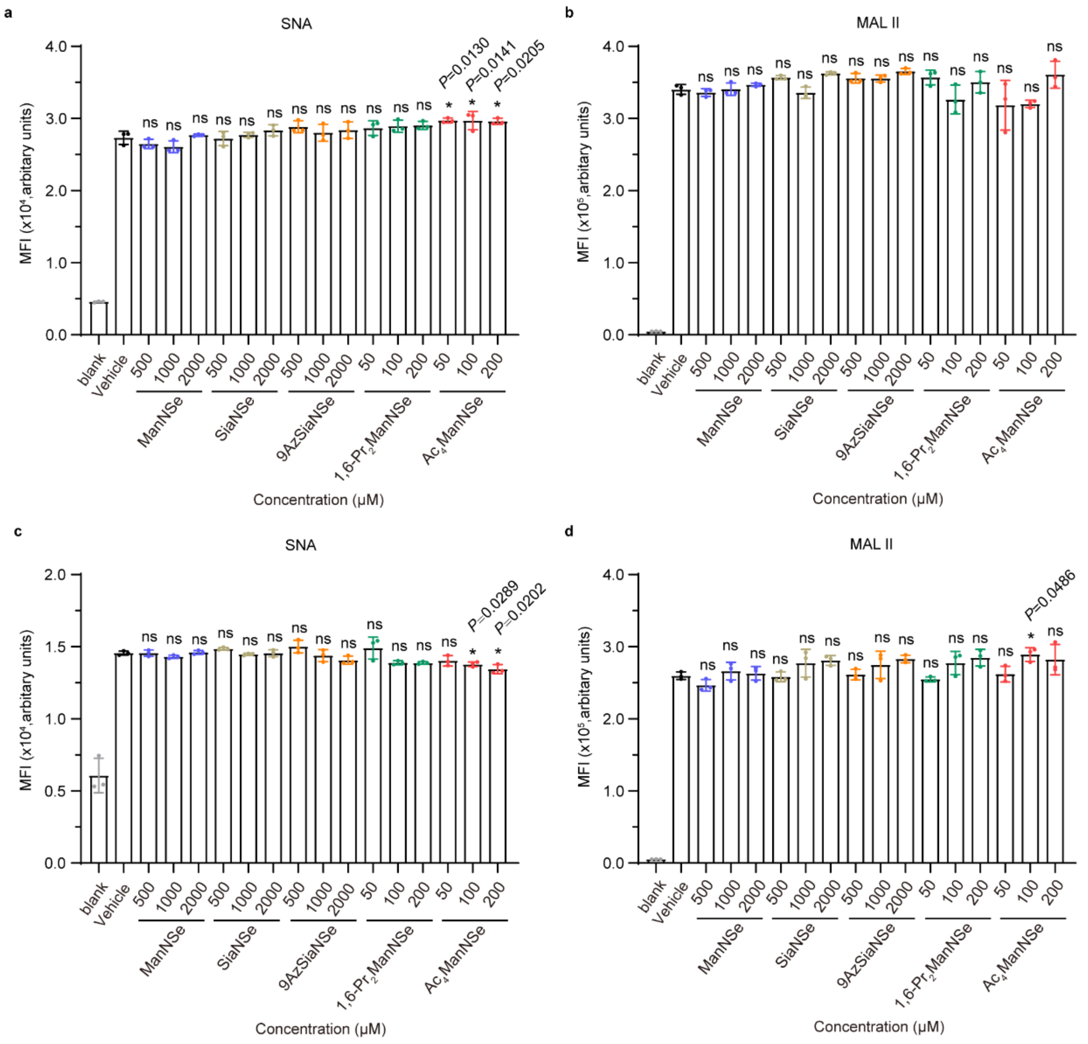

Supplementary Fig. 2 Flow cytometry analysis of sialic acid levels of SeMOE-treated cells. α2,6-linked and α2,3-linked sialic acids were recognized by Sambucus nigra (SNA) and Maackia amurensis leukoagglutinin II(MALII), respectively. **a-b**, Flow cytometry analysis of α2,6-linked sialic acids (**a**) and α2,3-linked sialic acids (**b**) of SeMOE-treated HeLa cells. **c-d**, Flow cytometry analysis of α2,6-linked sialic acids (**c**) and α2,3-linked sialic acids (**d**) of SeMOE-treated MCF-7 cells. Error bars represent mean ± SD of n= 3 independent biological replicates. *P < 0.05, **P < 0.01, ***P < 0.001, ns, not significant (one-way ANOVA followed by post hoc Dunnett's test). Source data including exact P values are provided as a Source Data file.

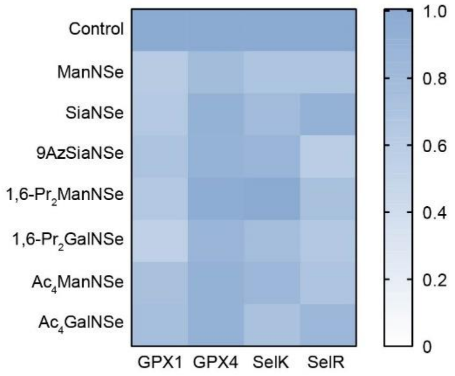

Supplementary Fig. 3 Selenoprotein mRNA levels of SeMOE-treated cells. HeLa cells were treated with PBS, 2 mM ManNSe, 2 mM SiaNSe, 2 mM 9AzSiaNSe, 200 μM 1,6-Pr_2ManNSe, 200 μM 1,6-Pr_2GalNSe, 100 μM Ac_4ManNSe or 100 μM Ac_4GalNSe for 48 h, respectively, followed by RT-qPCR analysis. n=4. Source data are provided as a Source Data file.

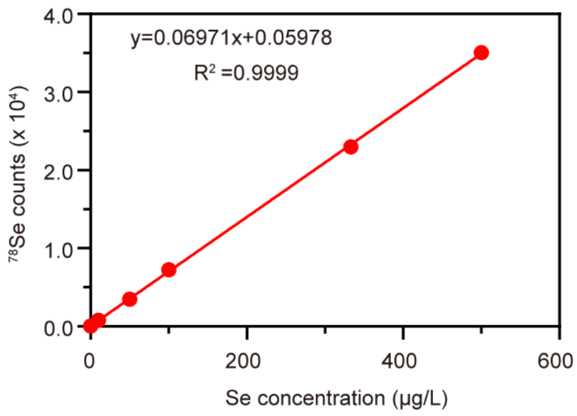

Supplementary Fig. 4 Calibration curve of Se standard solution. The calibration curve was constructed by Se standard solution of 0, 1, 10, 50, 100, 333 and 500 ppb (μg/L), respectively, against ^{78}Se-signal intensity, on the solution nebulization ICP-MS (PerkinElmer, NexION 300D, USA). Error bars represent mean ± SD of n= 3 independent biological replicates. Source data are provided as a Source Data file.

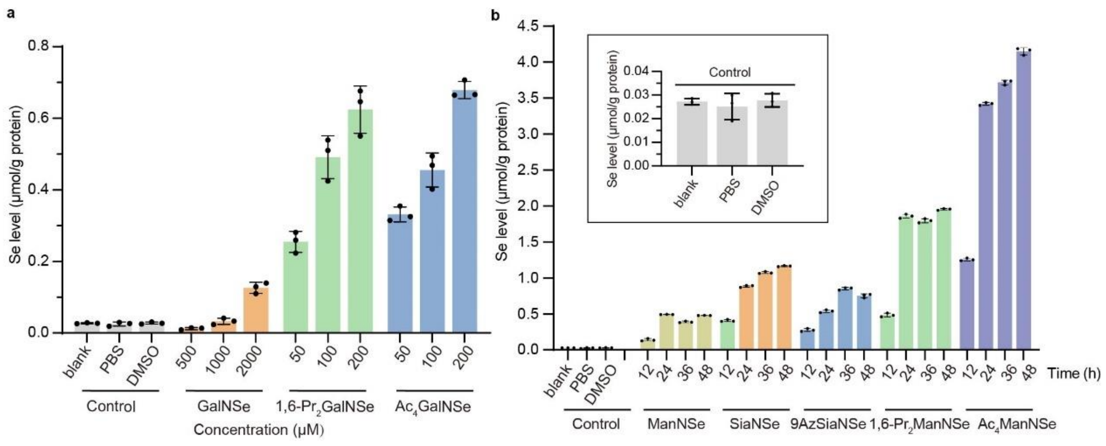

Supplementary Fig. 5 Se levels of glycoproteins from SeMOE-treated HeLa cells. **a,** Se levels of glycoproteins from HeLa cells treated with GalNSe, 1,6-Pr_2GalNSe or Ac_4GalNSe at varied concentrations for 48 h. **b,** Se levels of glycoproteins from HeLa cells treated with vehicle, 2 mM ManNSe, 2 mM SiaNSe, 2 mM 9AzSiaNSe, 200 μM 1,6-Pr_2ManNSe and 100 μM Ac_4ManNSe for the indicated time, respectively. Error bars represent mean ± SD of n= 3 independent biological replicates. Source data are provided as a Source Data file.

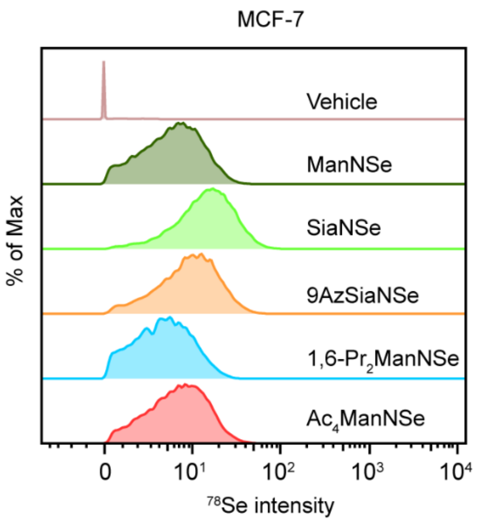

Supplementary Fig. 6 CyTOF analysis of SeMOE-treated MCF-7 cells. MCF-7 cells were treated with vehicle, 2 mM ManNSe, 2 mM SiaNSe, 2 mM 9AzSiaNSe, 200 µM 1,6-Pr_2ManNSe, and 100 µM Ac_4ManNSe for 48 h, respectively, followed by CyTOF analysis. *m/z* at 78 was used for calculation of ^{78}Se. At least 30,000 events were gated and analyzed according to the CyTOF gating strategy (Supplementary Note 3).

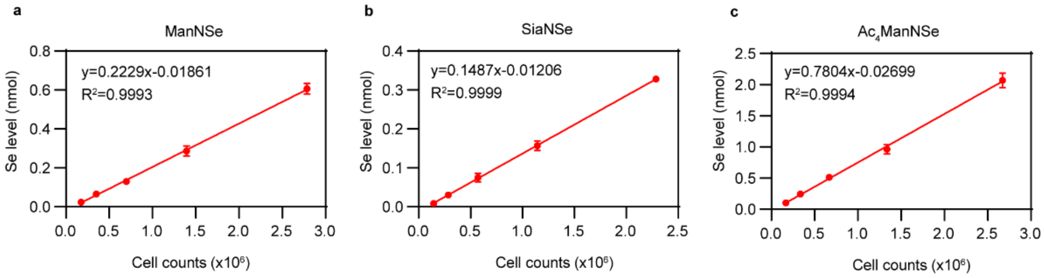

Supplementary Fig. 7 Linear correlation between cell counts and Se level. HeLa cells were treated with 2 mM ManNSe **(a)**, 2 mM SiaNSe **(b)** or 100 µM Ac4ManNSe and **(c)** for 48 h, respectively. Cells were trypsinized, separated based on cell counting numbers ranging from 0 - 2.7 × 10^6 as various groups. Each group was then subjected to ICP-MS analysis. The linear regression for SeMOE probes is shown in each chart. Error bars represent mean ± SD of n= 3 independent biological replicates. Source data are provided as a Source Data file.

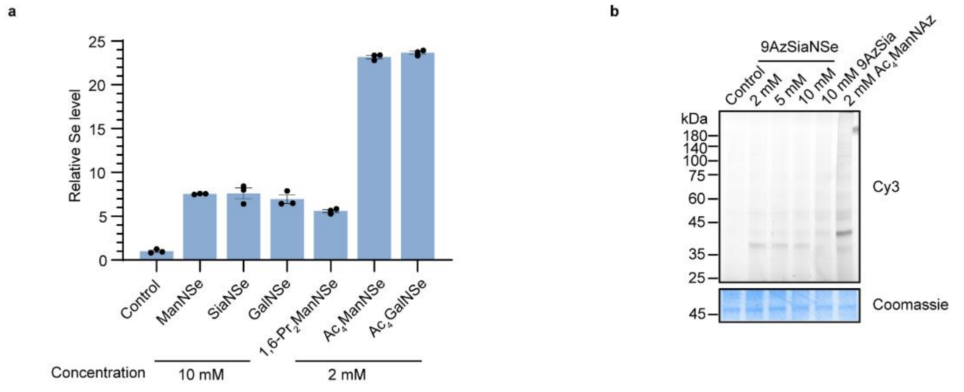

Supplementary Fig. 8 S-glyco-modification of SeMOE probes. **a,** Relative Se level of HeLa cell lysates treated with respective SeMOE probes at varied concentrations for 2 h. **b,** In-gel fluorescence scanning showing HeLa cell lysates treated with 9AzSiaNSe at varied concentrations for 2 h, followed by reaction with alkyne-Cy3. Error bars represent mean ± SD of n= 3 independent biological replicates. Source data are provided as a Source Data file.

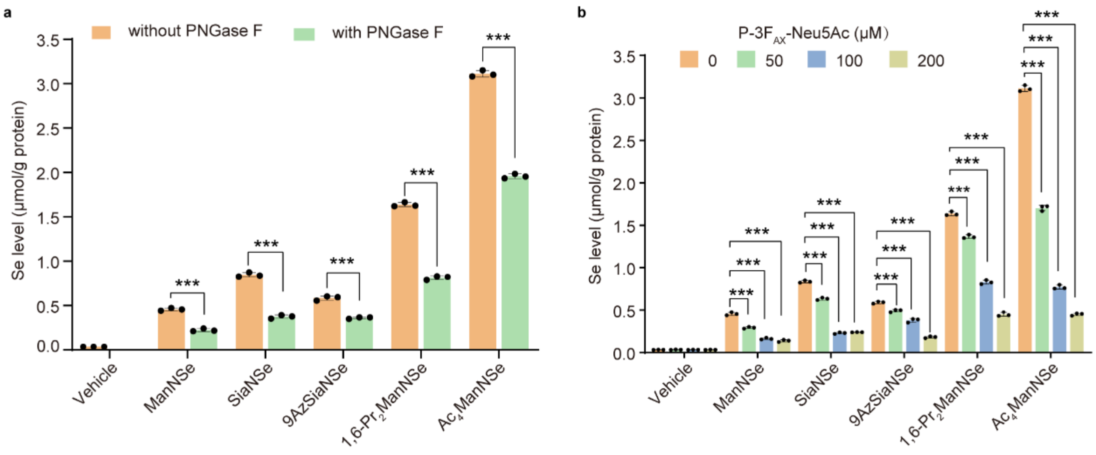

Supplementary Fig. 9 Se levels of glycoproteins from respective SeMOE probe-labelled HeLa cells treated with PNGase F or P-3F$_{AX}$-Neu5Ac. HeLa cells were treated with vehicle, 2 mM ManNSe, 2 mM SiaNSe, 2 mM 9AzSiaNSe, 200 μM 1,6-Pr$_2$ManNSe, and 100 μM Ac$_4$ManNSe for 48 h, respectively, then treated with or without PNGase F **(a)** or the indicated concentrations of P-3F$_{AX}$-Neu5Ac **(b)**, followed by ICP-MS analysis. Error bars represent mean ± SD of n= 3 independent biological replicates. *P < 0.05, **P < 0.01, ***P < 0.001, ns, not significant. Unpaired two-tailed t-test **(a)**. One-way ANOVA, post hoc Dunnett's test **(b)**. Source data including exact P values are provided as a Source Data file.

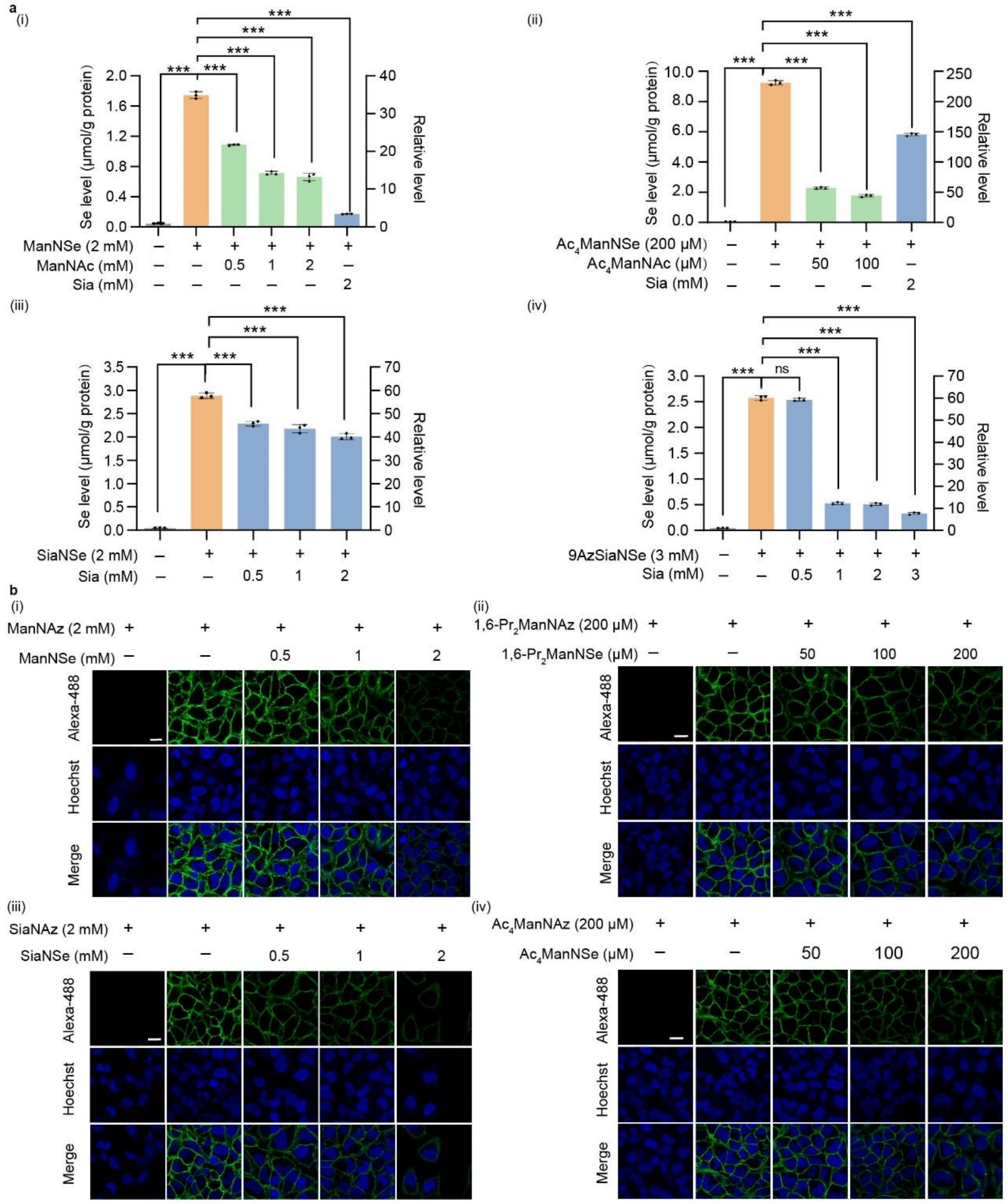

Supplementary Fig. 10 Metabolic competition between SeMOE probes and known monosaccharides involved in sialic acid metabolism. **a,** Se levels of glycoproteins from Hela cells treated with indicated SeMOE probes and known monosaccharides involved in sialic acid metabolism for 48 h. **b,** Confocal fluorescence imaging of HeLa cells treated with indicate selenium-based and azide-based unnatural monosaccharides for 48 h. Scale bar: 10 μm. Error bars represent mean ± SD of n= 3 independent biological replicates. **P* < 0.05, ***P* < 0.01, ****P* < 0.001, ns, not significant (One-way ANOVA, post hoc Dunnett's test). Source data including exact *P* values are provided as a Source Data file.

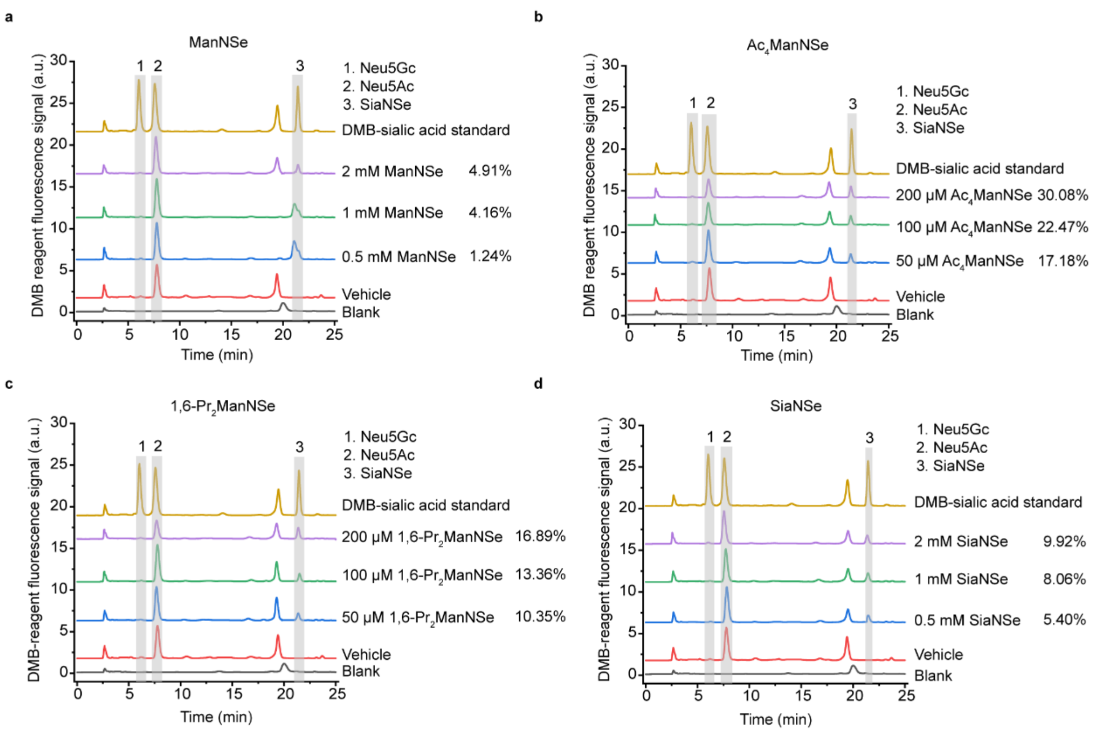

Supplementary Fig. 11 DMB-derived sialic acid analysis of SeMOE-treated HeLa cells. **a-d,** Glycoproteins from HeLa cells treated with indicated SeMOE probes for 48 h were isolated, and reacted with the fluorogenic 1,2-diamino-4,5-methylenedioxybenzene (DMB) probe. HPLC analysis quantified the presence and abundance of specific sialic acids. Peak 1, 2 and 3 represent Neu5Gc, Neu5Ac and SiaNSe, respectively. The metabolic incorporation rate is calculated as the peak area ratio between peak 3 and the sum of peak 1, 2, and 3. Source data are provided as a Source Data file.

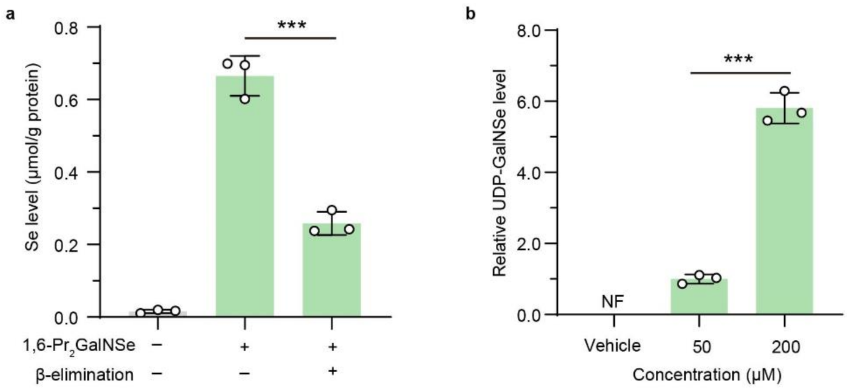

Supplementary Fig. 12 SeMOE of 1,6-Pr2GalNSe via GalNAc salvage pathway. a, MCF-7 cells were treated with vehicle (PBS) or 200 μM 1,6-Pr2GalNSe for 48 h, lysed for glycoprotein extraction, followed by β-elimination treatment and ICP-MS analysis. **b,** MCF-7 cells were treated with vehicle (PBS) or 1, 6-Pr2GalNSe for 48 h. UPLC-TSQ-MS/MS was performed to determine the cellular level of UDP-GalNSe. NF, Not Found. Error bars represent mean ± SD of n= 3 independent biological replicates. **P* < 0.05, ***P* < 0.01, ****P* < 0.001, ns, not significant (two-tailed Student's t-test). Source data including exact *P* values are provided as a Source Data file.

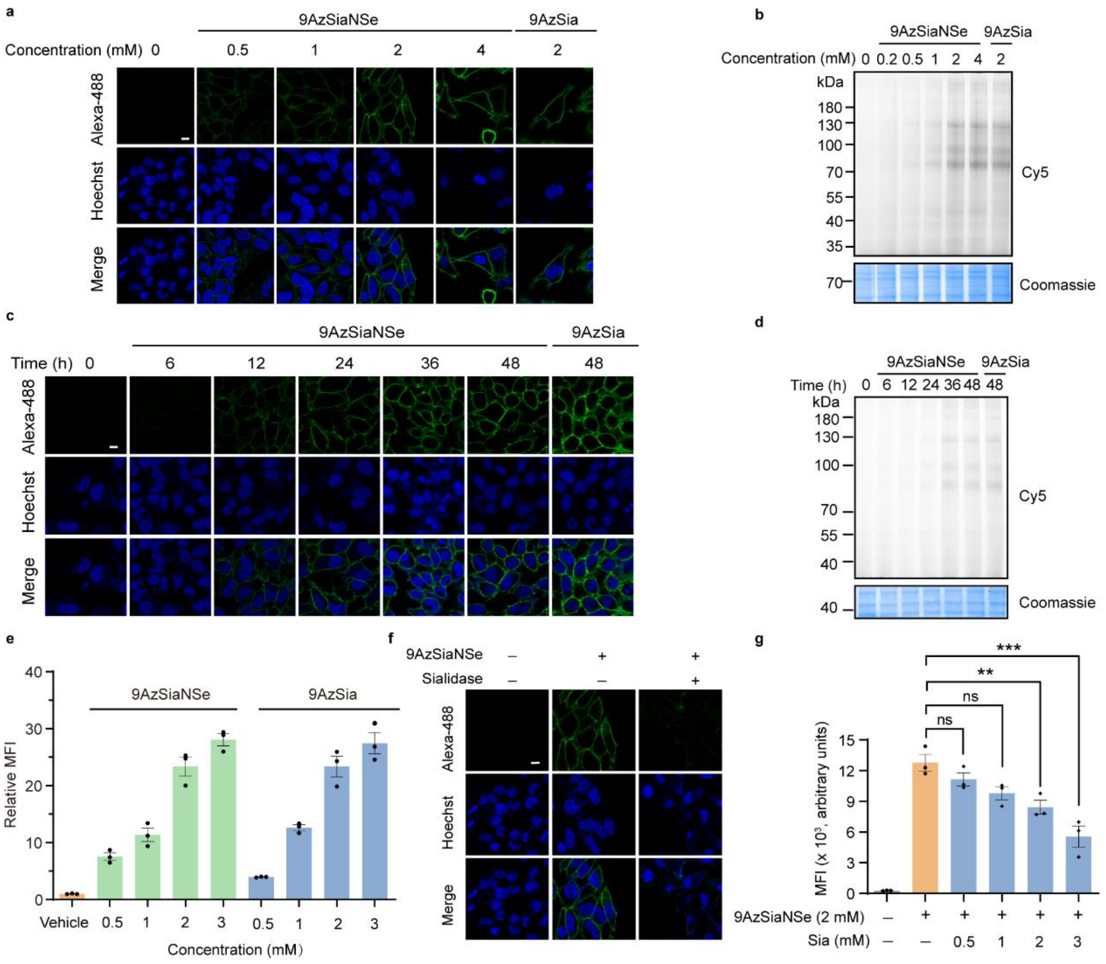

Supplementary Fig. 13 Metabolic labeling of HeLa cells by 9AzSiaNSe. **a-b,** Confocal fluorescence imaging **(a)** or in-gel fluorescence scanning **(b)** of HeLa cells treated with 9AzSiaNSe at indicated concentrations for 48 h, respectively. **c-d,** Confocal fluorescence imaging **(c)** or in-gel fluorescence scanning **(d)** of HeLa cells treated with 2 mM 9AzSiaNSe for indicated time. **e,** Flow cytometry analysis of 9AzSiaNSe-or 9AzSia-treated HeLa cells. **f,** Confocal fluorescence imaging of 9AzSiaNSe-labelled Hela cells treated with or without sialidase, followed by reaction with alkyne-Alexa 488. Scale bar: 10 µm. **g,** Flow cytometry analysis of HeLa cells treated with 9AzSiaNSe and Sia at indicated concentrations for 48 h. Error bars represent mean ± SD of n= 3 independent biological replicates. *P < 0.05, **P < 0.01, ***P < 0.001, ns, not significant (One-way ANOVA, post hoc Dunnett's test). Source data including P values are provided as a Source Data file.

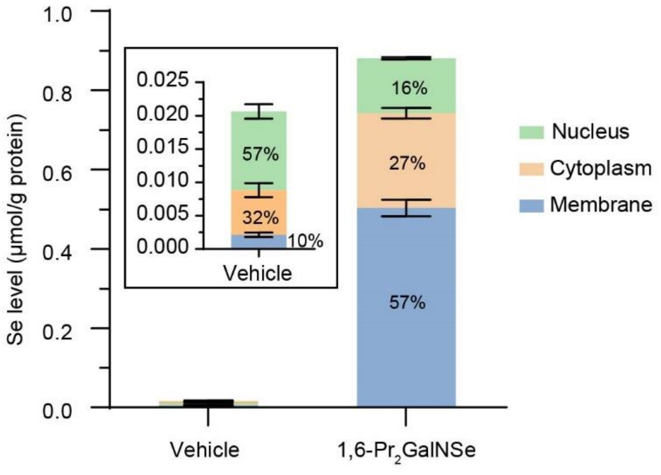

Supplementary Fig. 14 Se level of different subcellular proteins in MCF-7 cells. MCF-7 cells were treated with vehicle (PBS) or 200 μM 1,6-Pr_2GalNSe for 48 h. Proteins in different subcellular regions were isolated and precipitated, followed by ICP-MS analysis. Error bars represent mean ± SD of n= 3 independent biological replicates. Source data are provided as a Source Data file.

a

Number	Peptide	Glycan composition	Protein	Gene
#1	GYYJQSEAGSHTIQR	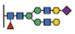	Q95604	HLA-C
#2	GYYJQSEAGSHTIQR	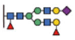	Q95604	HLA-C
#3	EAGJHTSGAGLVQIJK	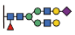	P20645	M6PR
#4	VTGLJCTTNHPINPK	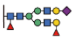	P08648	ITGA5
#5	AFJSTLPTMAQMEK	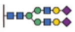	P16070	CD44
#6	AFJSTLPTMAQMEK	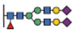	P16070	CD44
#7	GYYJQSEAGSHTIQR	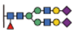	Q95604	HLA-C
#8	VTGLJCTTNHPINPK	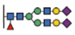	P08648	ITGA5
#9	KDFEDLYTPVJGSIVIVR	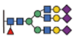	P02786	TFRC
#10	KEJSSEICSNNGECVCGQCVCR	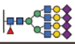	P05556	ITGB1

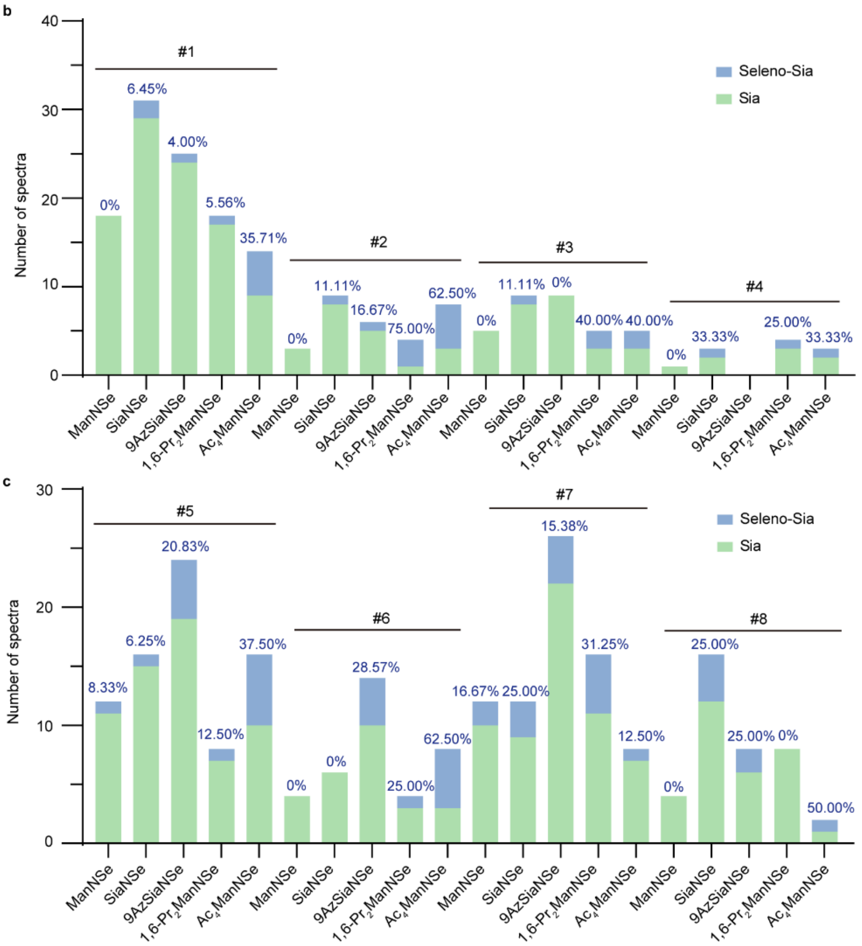

Supplementary Fig. 15 Examples of glycopeptides in pGlyco3 analysis. a, Peptide sequences and glycan compositions of ten random glycopepetides in common across all data sets in pGlyco3 analysis. **b-c**, Spectra numbers of seleno-Sia and natural Sia in glycopeptide #1 to #8. The percentages in blue font represent seleno-Sia incorporation rates of different glycopeptides. For glycopeptide #9 and #10, there are no seleno-sia were found (shown in source data). Source data are provided as a Source Data file.

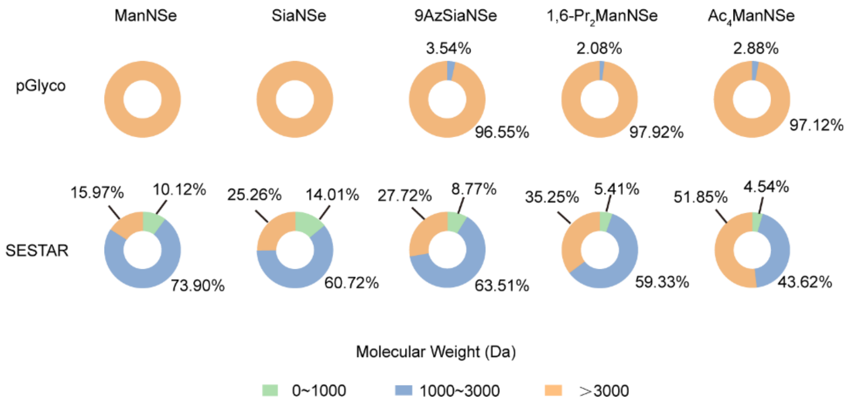

Supplementary Fig. 16 Comparison of the molecular weight of glycopeptides identified in pGlyco3 and SESTAR++ searches. Source data are provided as a Source Data file.

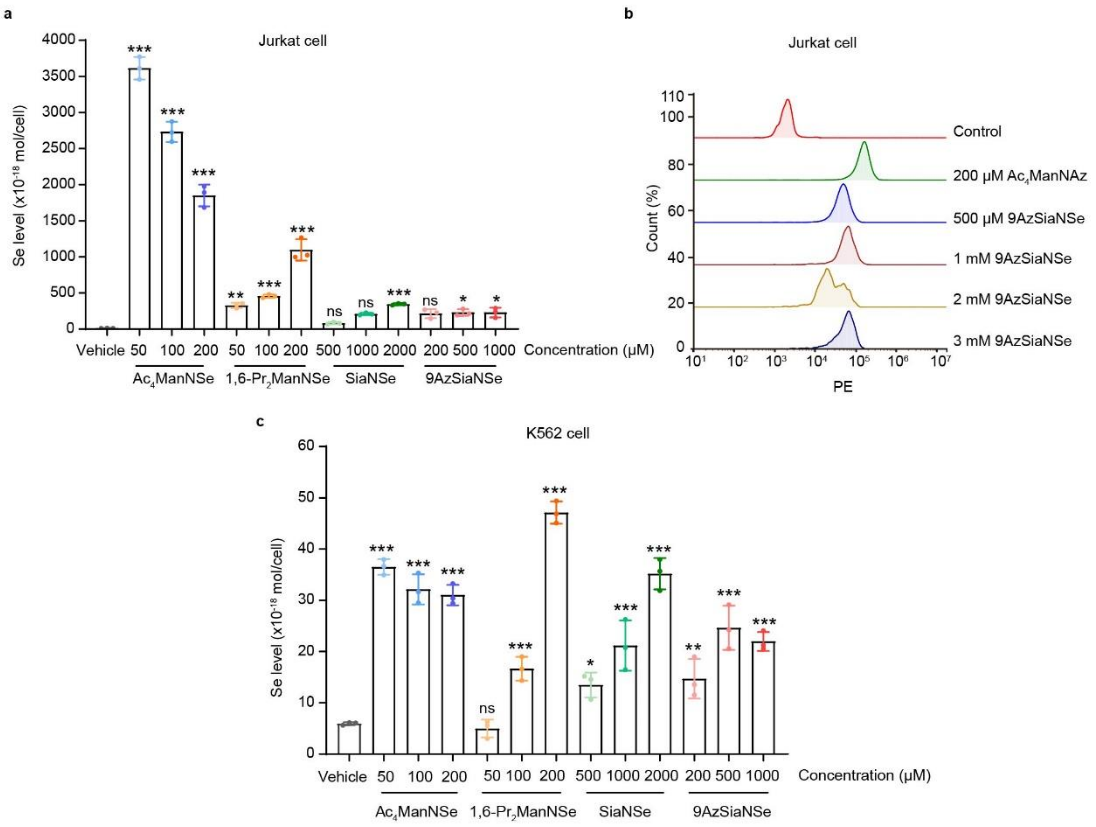

Supplementary Fig. 17 Applicability of SeMOE in Jurkat and K562 cells. a, Se levels of Jurkat cells treated with respective SeMOE probes at indicated concentrations for 48 h were measured by ICP-MS. **b,** Flow cytometry analysis of 9AzSiaNSe-labelled Jurkat cells, followed by reaction with alkyne-Alexa 488. **c,** Se levels of K562 cells treated with respective SeMOE probes at indicated concentrations for 48 h. Error bars represent mean ± SD of n= 3 independent biological replicates. **P* < 0.05, ***P* < 0.01, ****P* < 0.001, ns, not significant (One-way ANOVA, post hoc Dunnett's test, compared with the vehicle group). Source data including *P* values are provided as a Source Data file.

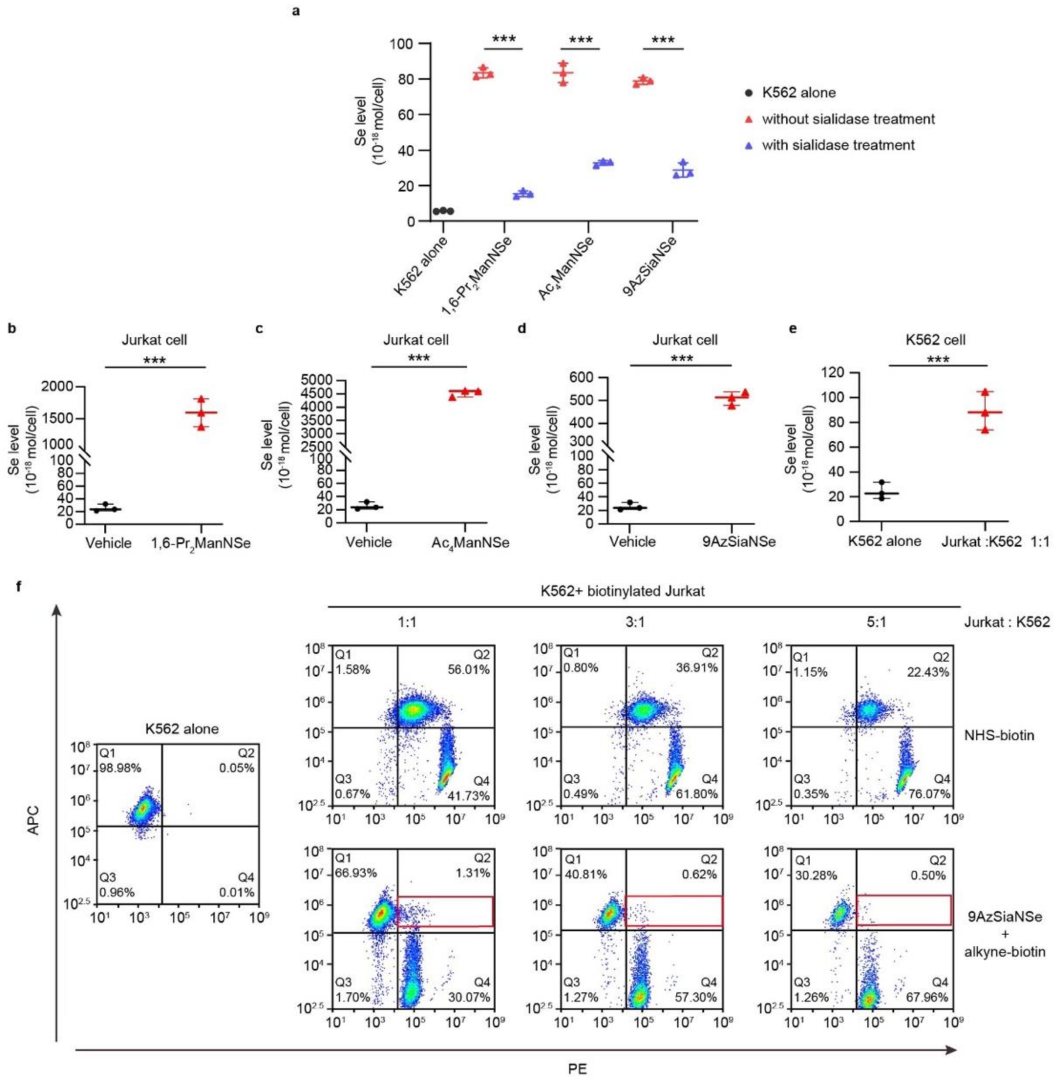

Supplementary Fig. 18 Glycan transfer quantification during Jurkat cell-K562 cell trogocytosis. a, Seleno-sialic acid transfer from SeMOE probe-labelled Jurkat cells to K562 cells during trogocytosis with or without sialidase treatment. **b-c,** Se levels of 50 μM $Ac_4ManNSe$-treated **(b)** or 200 μM 1,6-$Pr_2ManNSe$-treated **(c)** Jurkat cells. **d,** Se levels of 1 mM 9AzSiaNSe-treated Jurkat cells. **e,** Seleno-sialic acid transfer from 9AzSiaNSe-labelled Jurkat cells to K562 cells during trogocytosis. **f,** Flow cytometry analysis of protein and sialic acid transfer during Jurkat and K562 cell trogocytosis. Jurkat cells treated with or without 9AzSiaNSe were biotinylated by NHS-biotin or alkyne-biotin, respectively. Did-APC stained K562 cells were co-incubated with biotinylated Jurkat cells in varied ratios at 37°C for 2 h, followed by incubation with streptavidin-PE at 4°C for 0.5 min. Error bars represent mean ± SD of n= 3 independent biological replicates. *P < 0.05, **P < 0.01, ***P < 0.001, ns, not significant (two-tailed Student's t-test). Source data including P values are provided as a Source Data file.

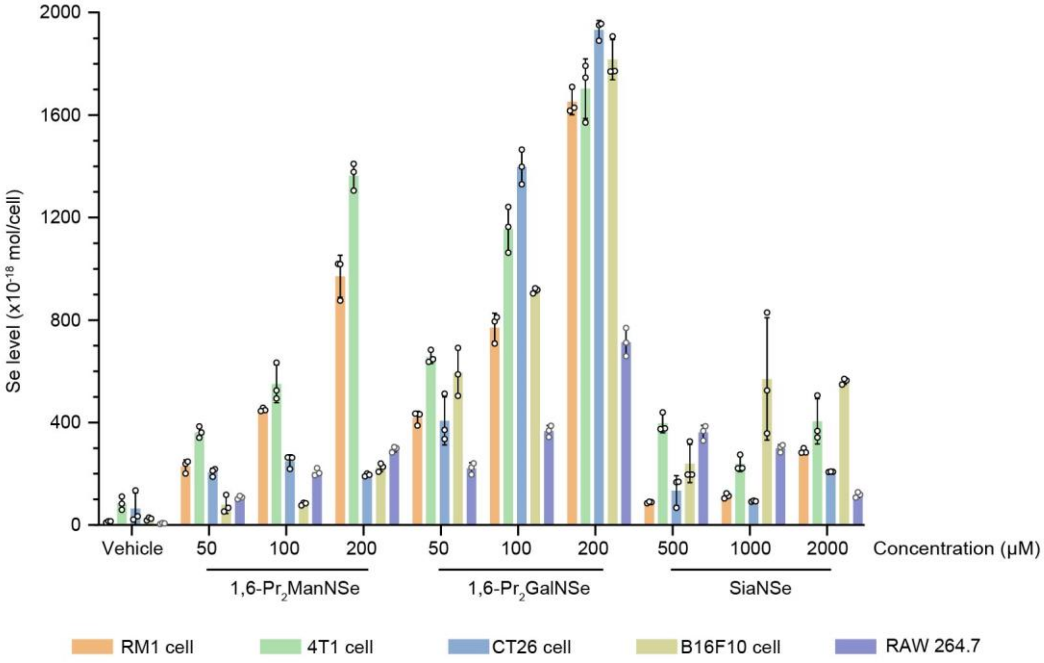

Supplementary Fig. 19 Systematic evaluation of SeMOE in various cancer cell lines. The cancer cells were treated with respective SeMOE probes at indicated concentrations for 48 h, and analyzed by ICP-MS. Error bars represent mean ± SD of n= 3 independent biological replicates. Source data are provided as a Source Data file.

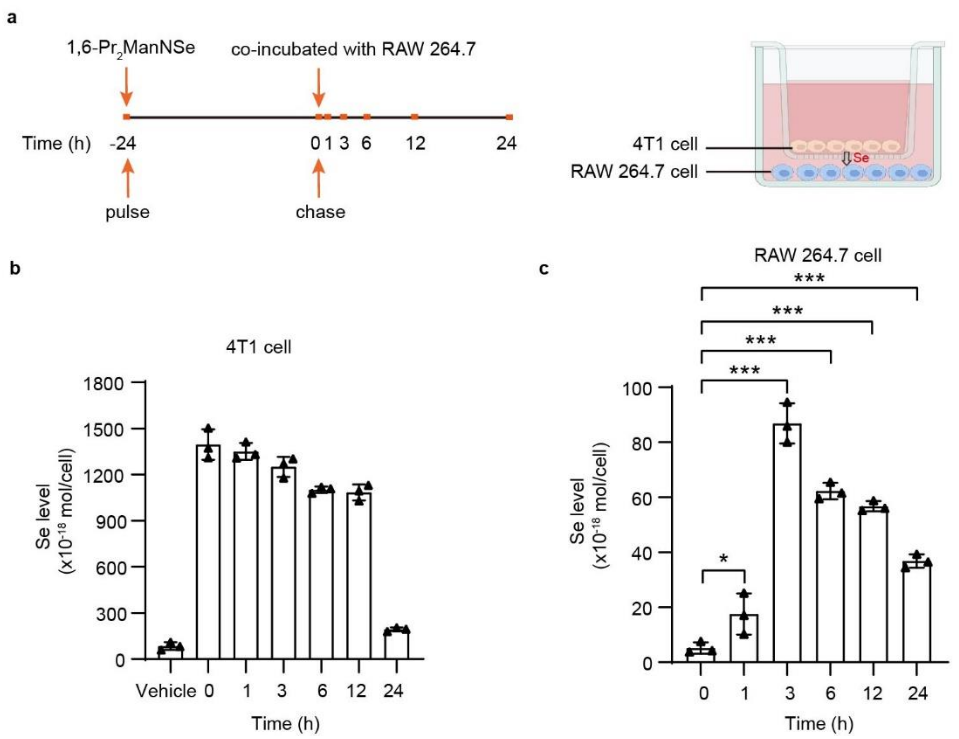

Supplementary Fig. 20 Seleno-sialic acid transfer assay during 4T1 and RAW 264.7 cells co-incubation. a, Schematic workflow of the pulse-chase experiment. 4T1 cells were treated with 200 μM 1,6-Pr_2ManNSe for 24 h, washed with PBS, and then co-incubated with RAW 264.7 cells in a 0.4 μm-sized transwell culture system for varied time. Se level of cancer cells and RAW 264.7 cells were analyzed by ICP-MS, respectively. Figure created with BioRender.com. **b,** Se levels of 4T1 cells during incubation with RAW 264.7 cells. **c,** Se transfer from 4T1 cells to RAW 264.7 cells. 0 h represents the initiation of co-incubation and chase experiment. Error bars represent mean ± SD of n= 3 independent biological replicates. *$P < 0.05$, **$P < 0.01$, ***$P < 0.001$, ns, not significant (one-way ANOVA followed by post hoc Dunnett's test). Source data including *P* values are provided as a Source Data file.

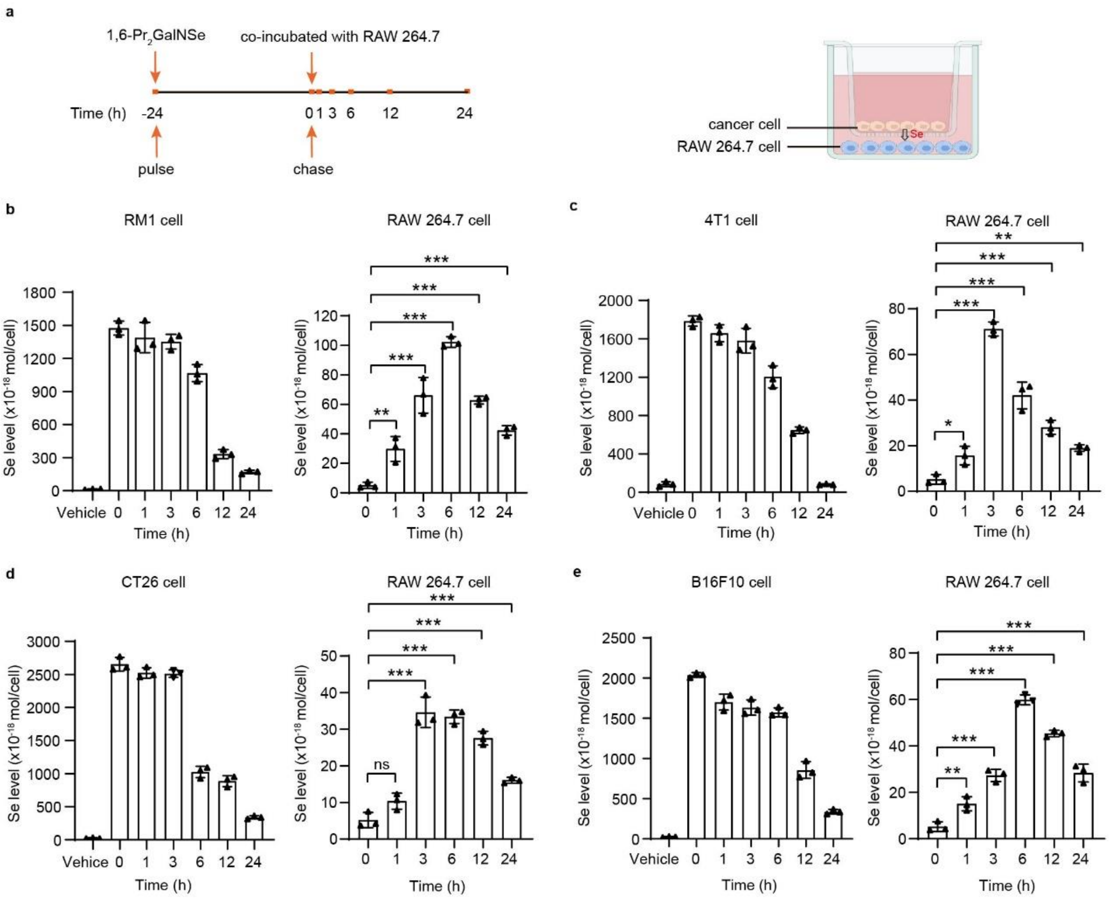

Supplementary Fig. 21 Selenoglycan transfer during cancer cell and RAW 264.7 cell transwell co-incubation. a, Workflow of the co-incubation of 1,6-Pr$_2$GalNSe-treated cancer cells and RAW 264.7 cells. Cancer cells were treated with 200 μM 1,6-Pr$_2$GalNSe for 24 h, washed, and then co-incubated with RAW 264.7 cells in a 0.4 μm-sized transwell culture system for varied time. Figure created with BioRender.com. **b-e,** Se levels of RM1 cells. **(b)**, 4T1 cells **(c)**, CT26 cells **(d)** and B16F10 cells **(e)** during incubation with RAW 264.7 cells, and selenoglycan transfer from cancer cells to RAW 264.7 cells. Error bars represent mean ± SD of n= 3 independent biological replicates. **P* < 0.05, ***P* < 0.01, ****P* < 0.001, ns, not significant (one-way ANOVA followed by post hoc Dunnett’s test). Source data including *P* values are provided as a Source Data file.

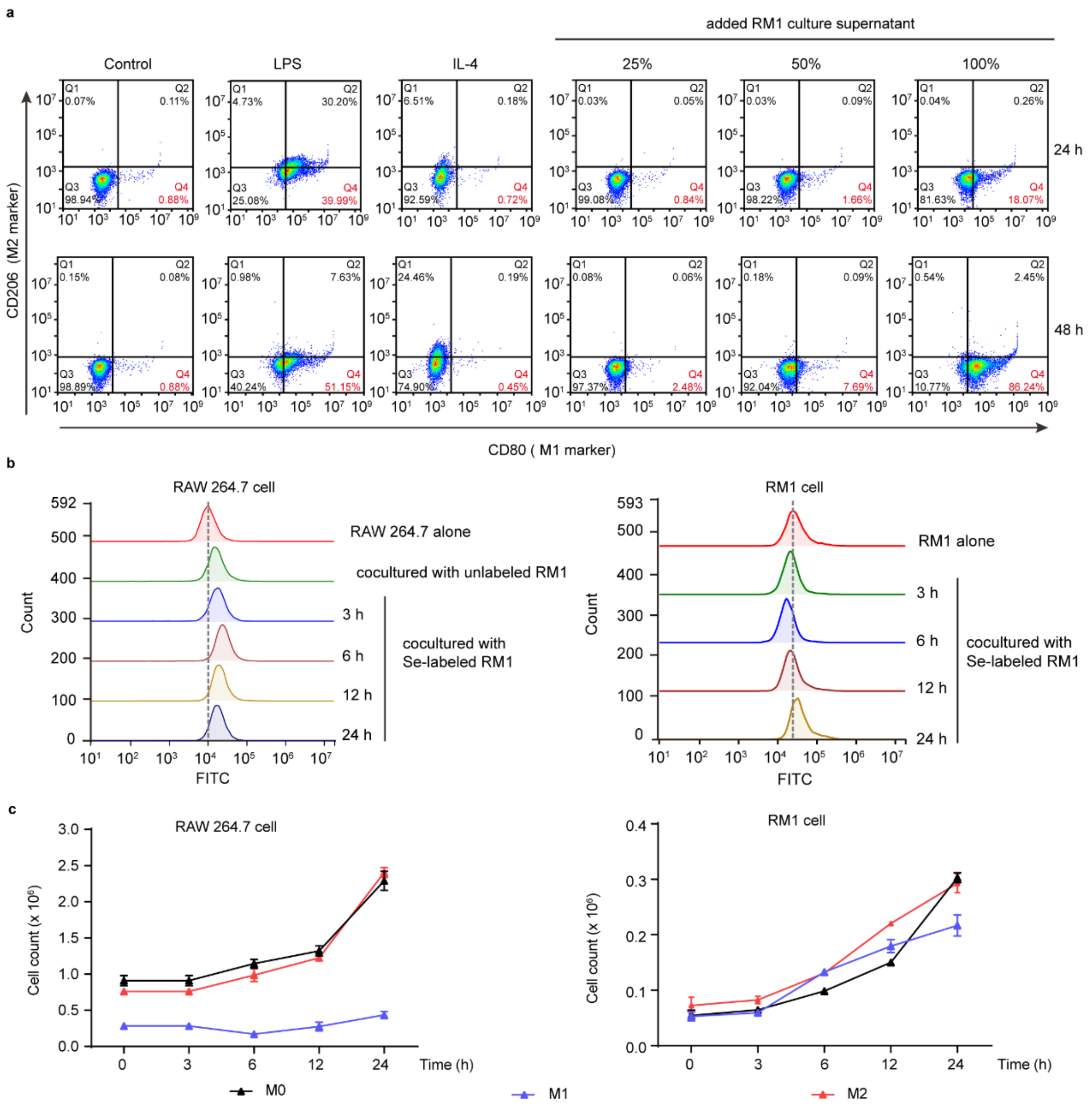

Supplementary Fig. 22 Biological effects of co-incubation of cancer cells and RAW 264.7 cells. a, RM1 cell culture supernatant-induced M1 polarization of RAW 264.7 cells. **b,** ROS assay of RAW 264.7 cells and RM1 cells during co-incubation. **c,** Cell counts of RAW 264.7 cells in different polarization states and RM1 cells during co-incubation. Error bars represent mean ± SD of n= 3 independent biological replicates. Source data are provided as a Source Data file.

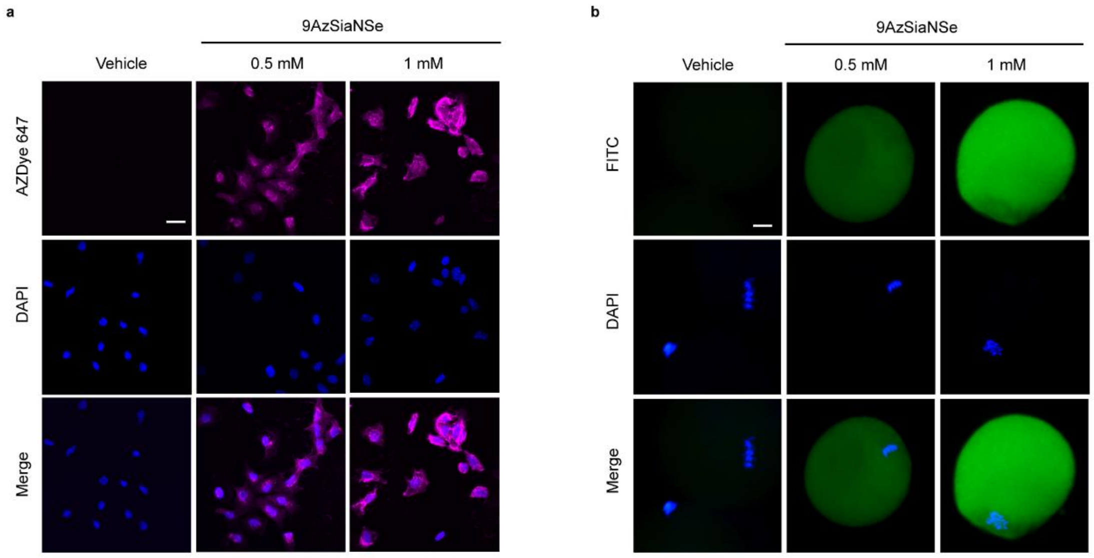

Supplementary Fig. 23 Confocal fluorescence imaging of 9AzSiaNSe-labelled GCs (a) and the oocytes after co-incubation with 9AzSiaNSe-labelled GCs (b). **a,** Mouse GCs were treated with vehicle (PBS) or 9AzSiaNSe at indicated concentrations for 48 h, respectively. The GCs were reacted with alkyne-AZDye 647 via click chemistry. **b,** Mouse primary oocytes were co-incubated with 9AzSiaNSe-labelled or PBS-treated GCs for 14-16 h, respectively. The oocytes were reacted with alkyne-AZDye 488 via click chemistry. The nucleus was stained by DAPI. Scale bar: 20 μm.

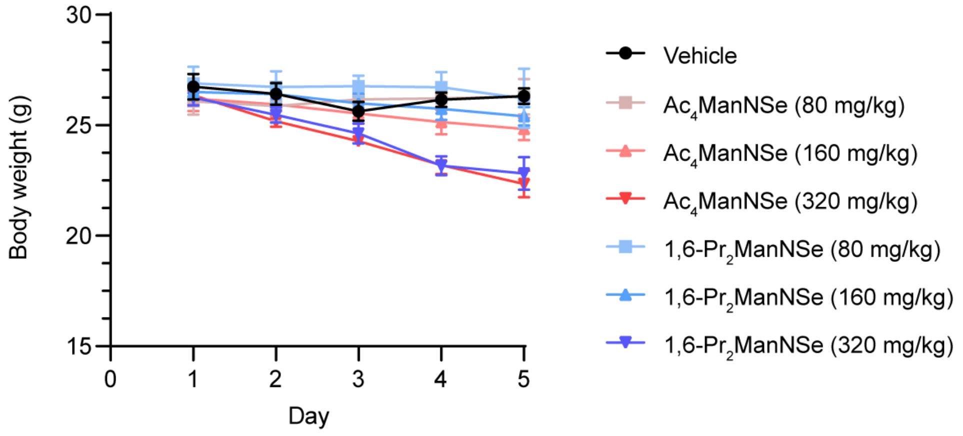

Supplementary Fig. 24 Body weight of SeMOE-treated BALB/c mice. Error bars represent mean ± SD of n= 3 independent biological replicates. Source data are provided as a Source Data file.

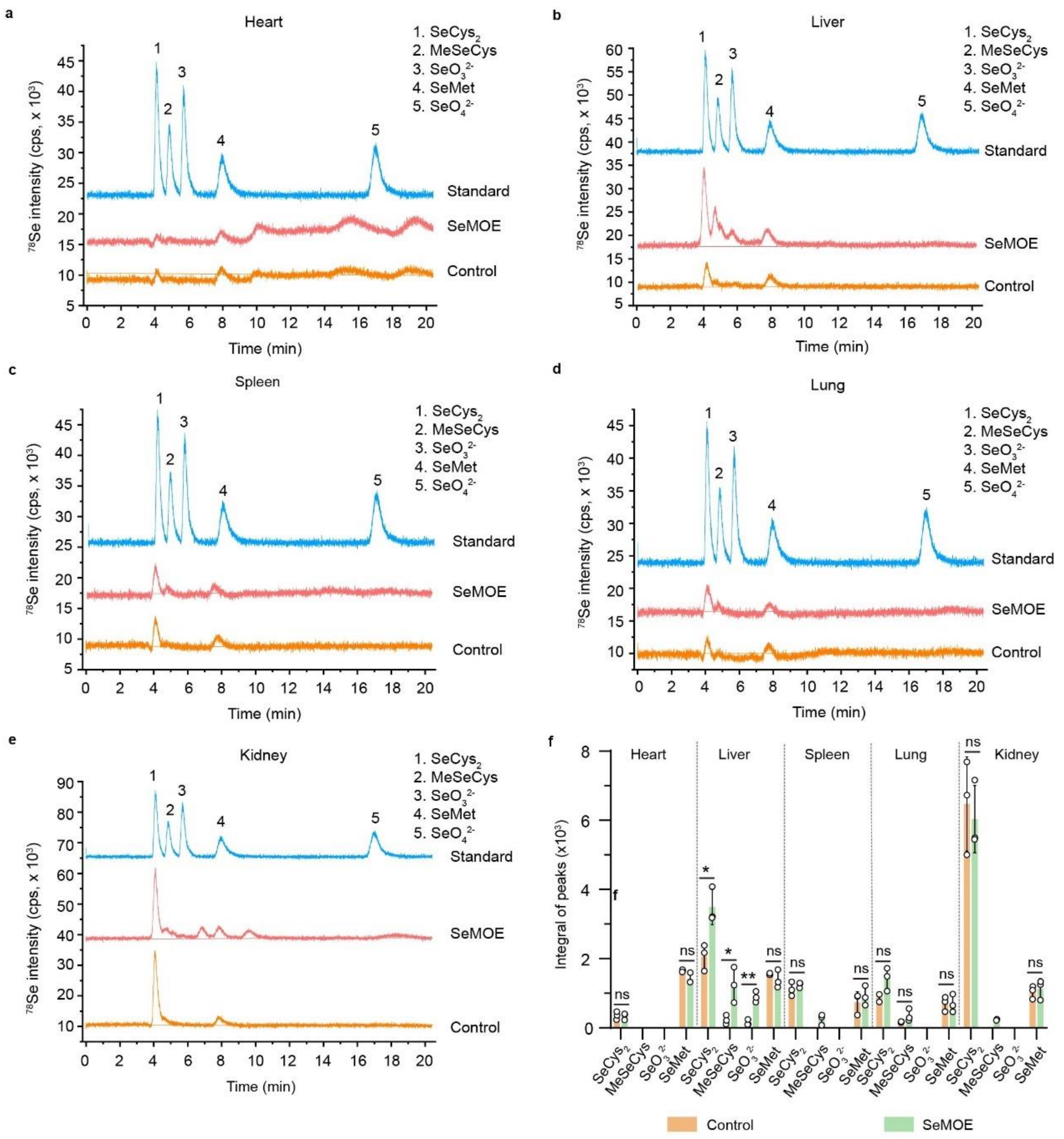

Supplementary Fig. 25 Selenoamino acid levels of SeMOE-treated mice. a-e, Male BALB/c mice (8 to 10 weeks old) were once-daily, intraperitoneally injected with Ac4ManNSe (160 mg Ac4ManNSe/kg/day), while control mice received the 70% DMSO alone. On day 5, mice were euthanized. The heart, liver, spleen, lung and kidney of mice were collected, digested by proteinase K and trypsin, followed by selenoamino acid analysis via ICP-MS. **f,** Comparison of the selenoamino acid peak integral between the Control group and SeMOE group. Error bars represent mean ± SD of n = 3 independent biological replicates. **P* < 0.05, ***P* < 0.01, ****P* < 0.001, ns, not significant (two-tailed Student's t-test). Source data are provided as a Source Data file.

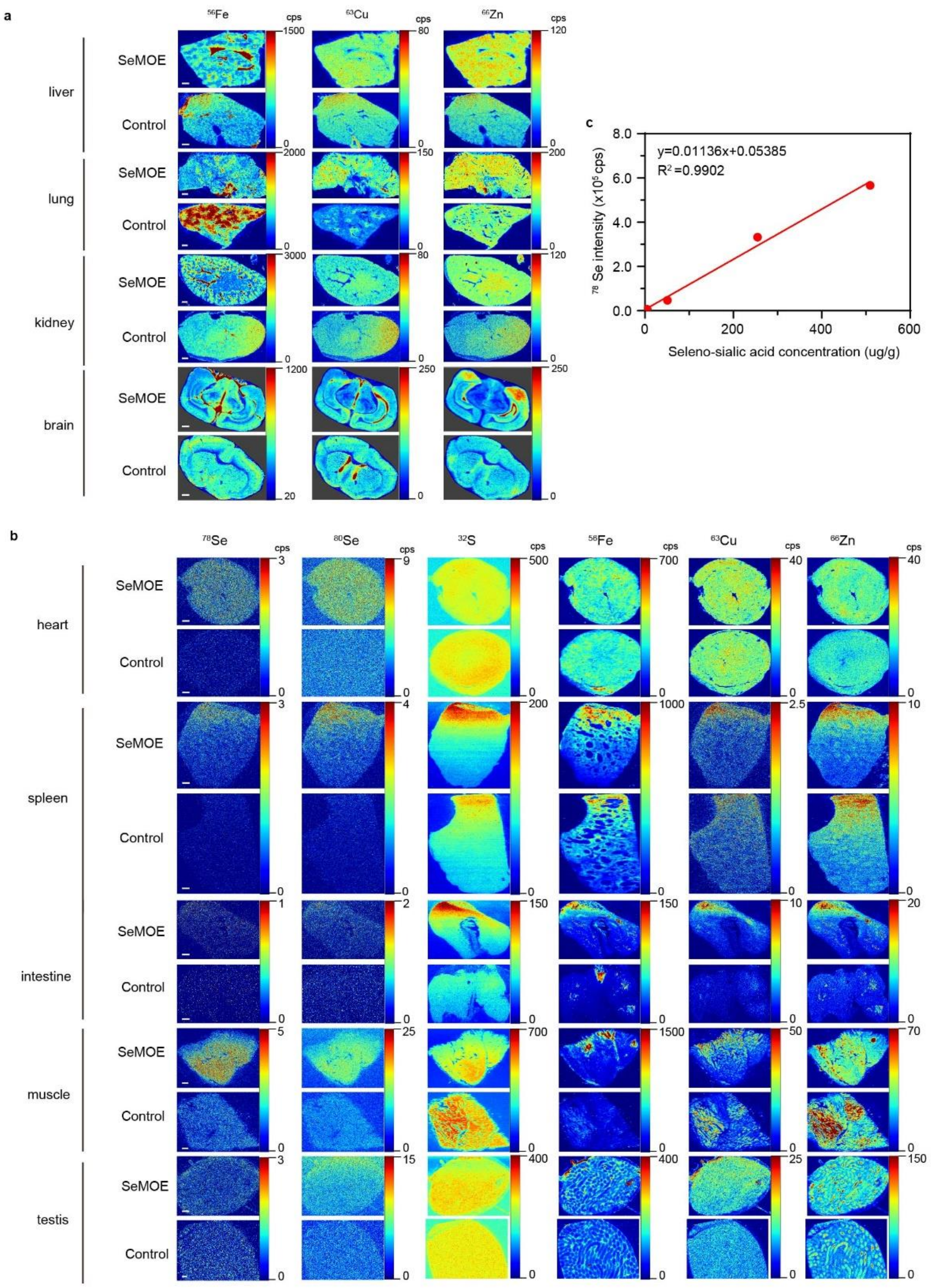

Supplementary Fig. 26 *In situ* visualization of sialoglycans in mouse tissues. **a,** *In situ* LA-ICP-MS imaging of ^{56}Fe, ^{63}Cu, and ^{66}Zn in mouse liver, lung, kidney and brain. **b,** *In situ* LA-ICP-MS imaging of sialoglycans in mouse heart, spleen, intestine, muscle and testis. Scale bar: 400 μm. **c,** Standard curve for *in situ* quantification of sialoglycans by LA-ICP-MS. The calibration curve was constructed by gelatin slices containing 0, 1, 10, 50, 100 ppm selenium, respectively, against ^{78}Se-signal intensity, on the LA-ICP-MS. Source data are provided as a Source Data file.

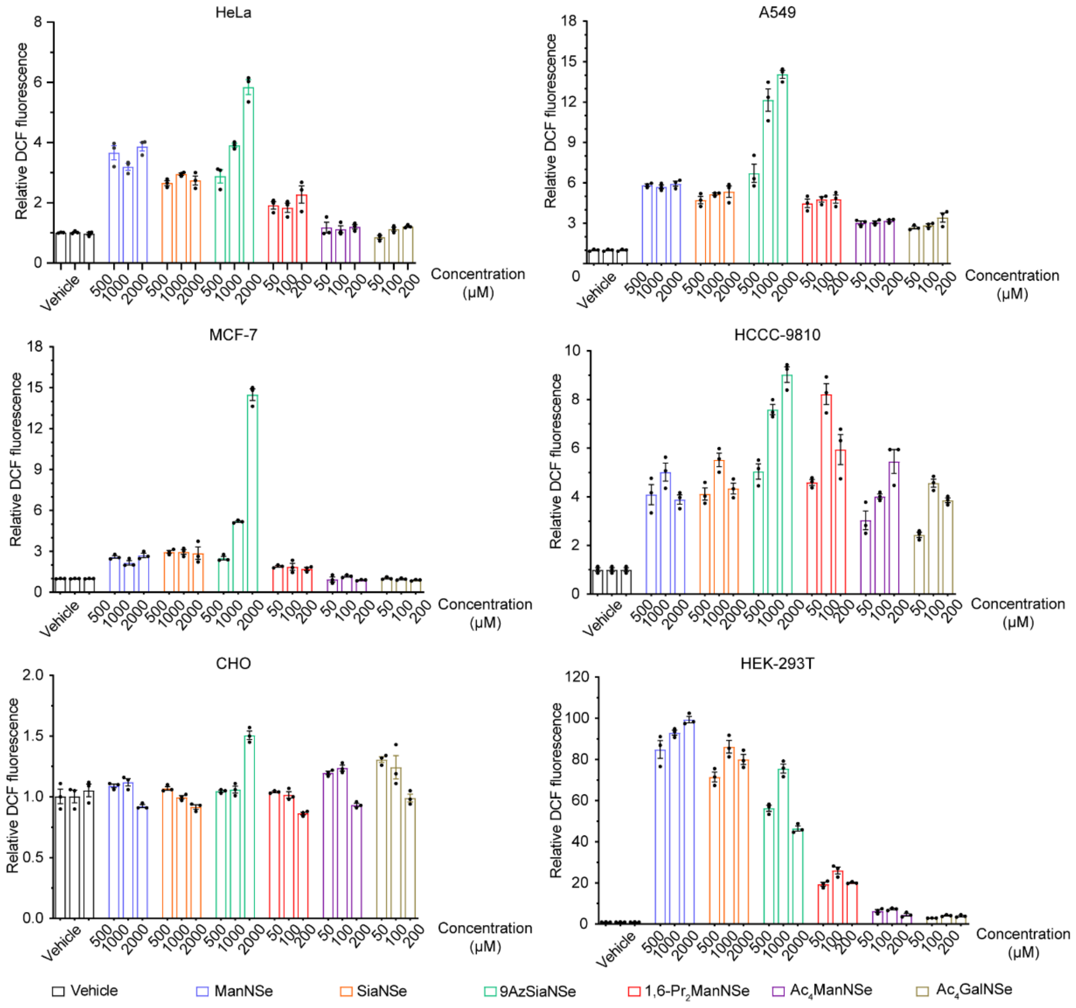

Supplementary Fig. 27 ROS assay of various cell lines treated with respective SeMOE probes at indicated concentrations for 24 h. The ROS level was assayed using flow cytometry. Error bars represent mean ± SD of n= 3 independent biological replicates. Source data including exact *P* values are provided as a Source Data file.

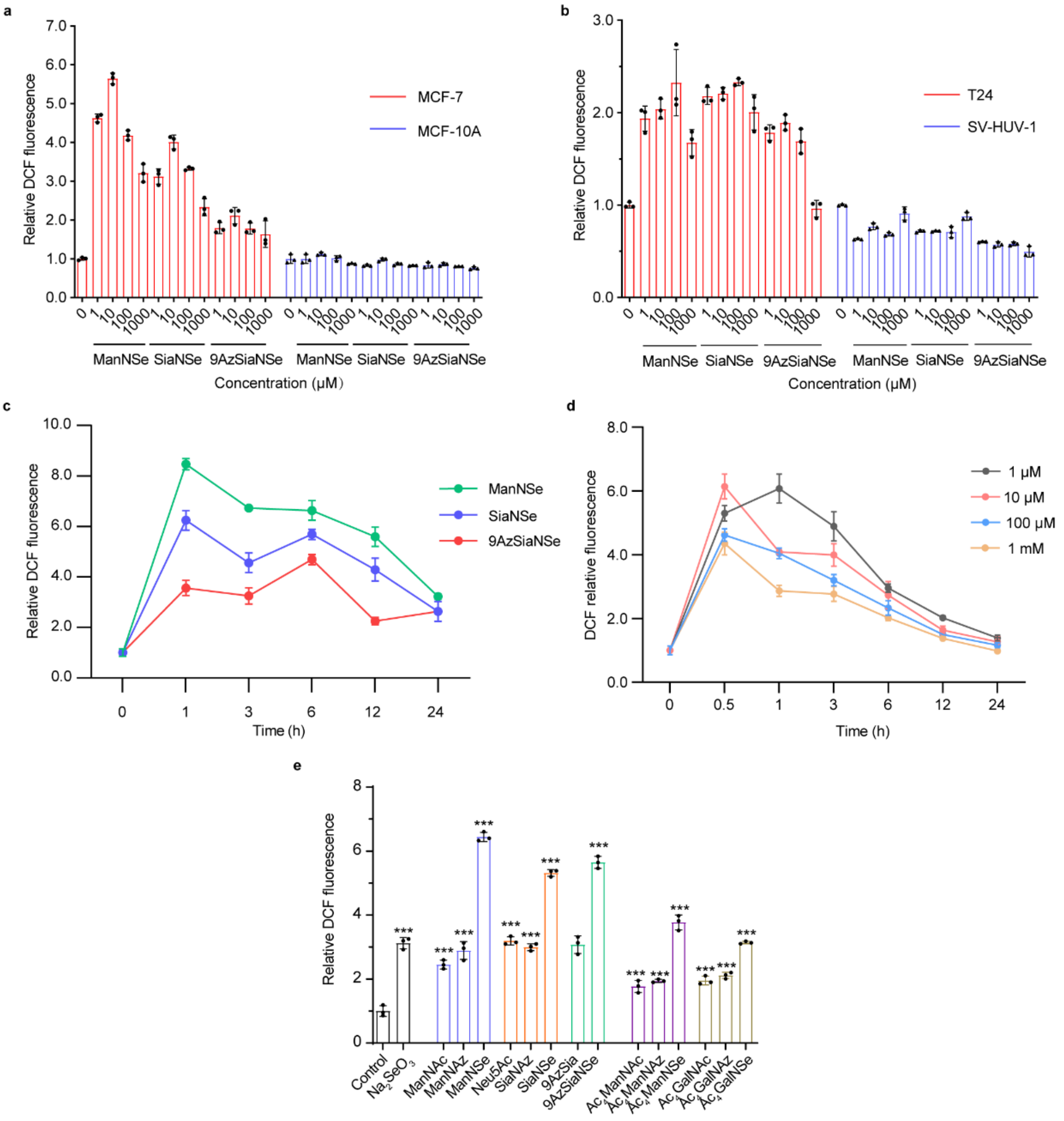

Supplementary Fig. 28 ROS assay of respective monosaccharide-treated cells. **a,** ROS assay of MCF-7 and MCF-10A cells treated with respective unnatural selenium-containing monosaccharides at indicated concentrations for 24 h. **b,** ROS assay of T24 and SV-HUV-1 cells treated with respective unnatural selenium-containing monosaccharides at indicated concentrations for 24 h. **c,** ROS assay of MCF-7 cells treated with 10 μM ManNSe, 10 μM SiaNSe or 10 μM 9AzSiaNSe for 1 h, respectively. **d,** ROS assay of MCF-7 cells treated with ManNSe at indicated concentrations for varies time. **e**, ROS assay of MCF-7 cells treated with 10 μM respective monosaccharides for 1 h. Error bars represent mean ± SD of n= 3 independent biological replicates. **P* < 0.05, ***P* < 0.01, ****P* < 0.001, ns, not significant (one-way ANOVA followed by post hoc Dunnett's test). Source data are provided as a Source Data file.

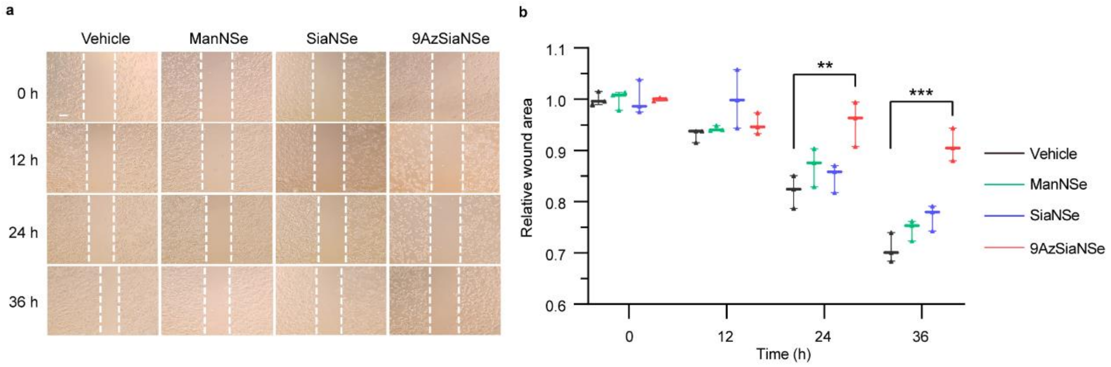

Supplementary Fig. 29 Inhibitory effects of 1 mM ManNSe, 1 mM SiaNSe or 1 mM 9AzSiaNSe on MCF-7 cell migration. Scale bar: 100 μm. Error bars represent mean ± SD of n = 3 independent biological replicates. *P < 0.05, **P < 0.01, ***P < 0.001, ns, not significant (one-way ANOVA followed by post hoc Dunnett's test). Source data including P values are provided as a Source Data file.

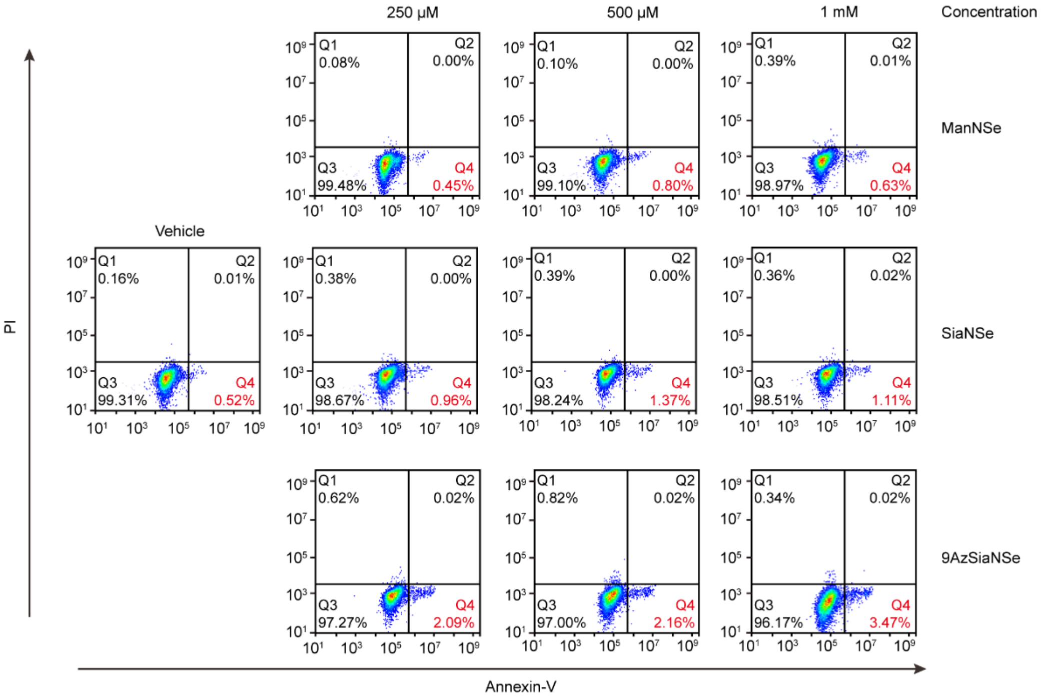

Supplementary Fig. 30 Apoptosis assay of MCF-7 cells after SeMOE treatment. MCF-7 cells were treated with ManNSe, SiaNSe, or 9AzSiaNSe at indicated concentrations for 48 h. The viability of each cell group was measured using a commercialized Annexin-V/PI assay. Source data are provided as a Source Data file.

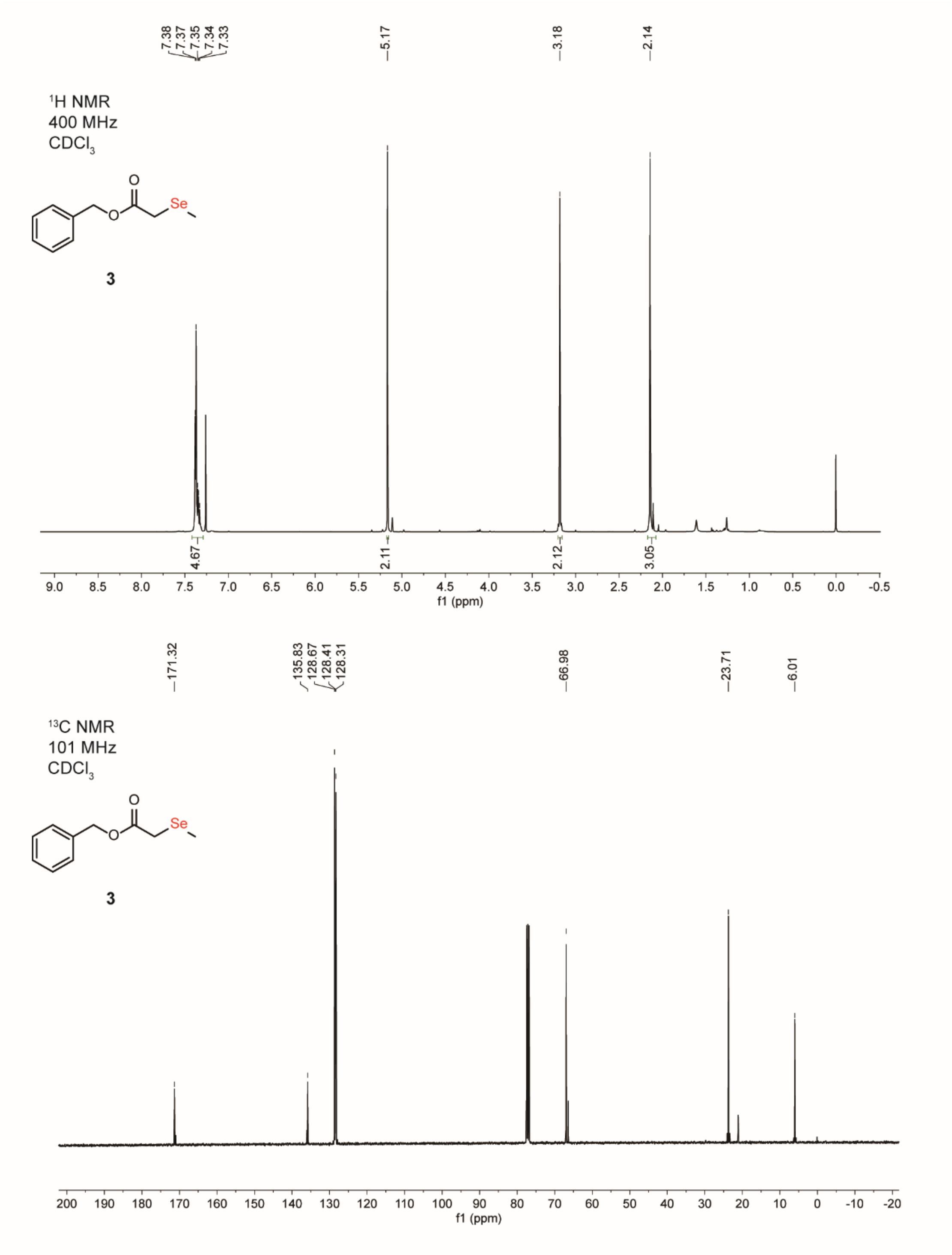

Supplementary Fig. 31 ^{1}H NMR and ^{13}C NMR spectrum of **3**

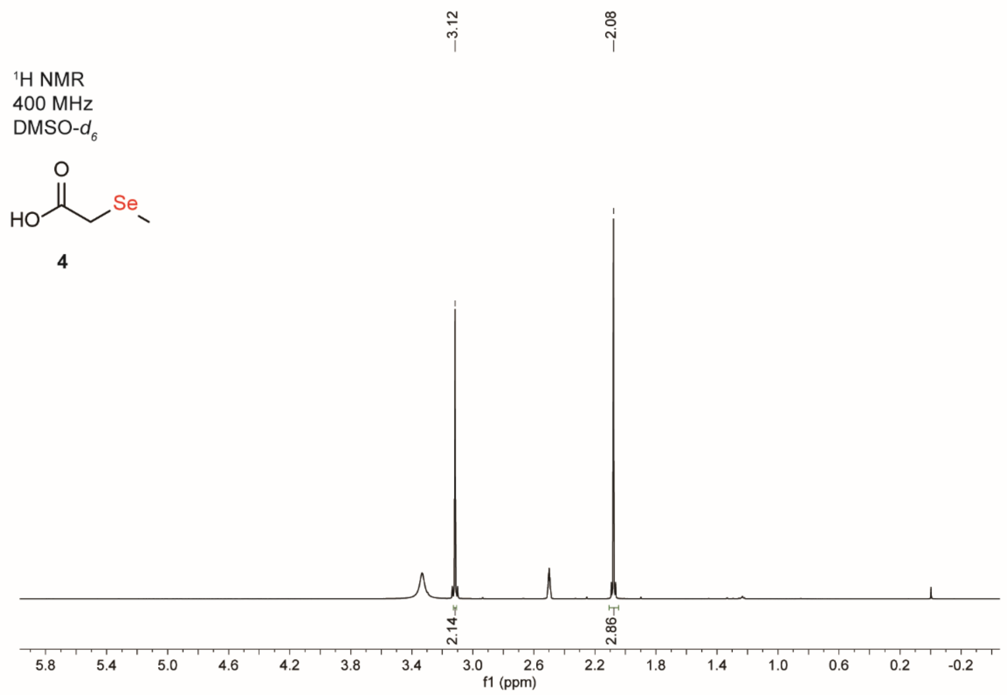

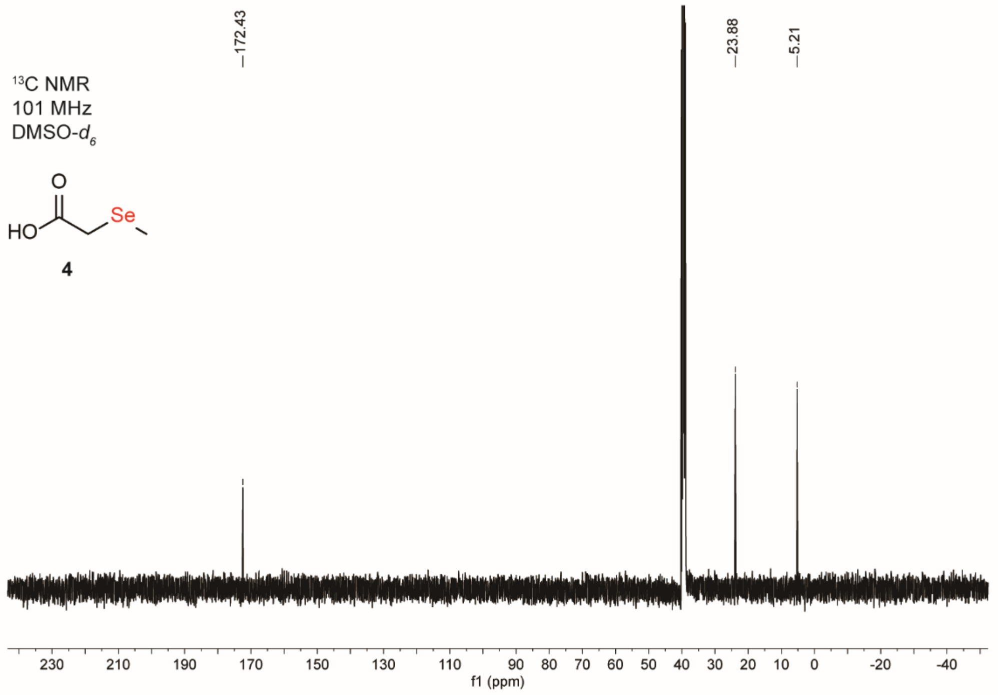

Supplementary Fig. 32 ^{1}H NMR and ^{13}C NMR spectrum of **4**

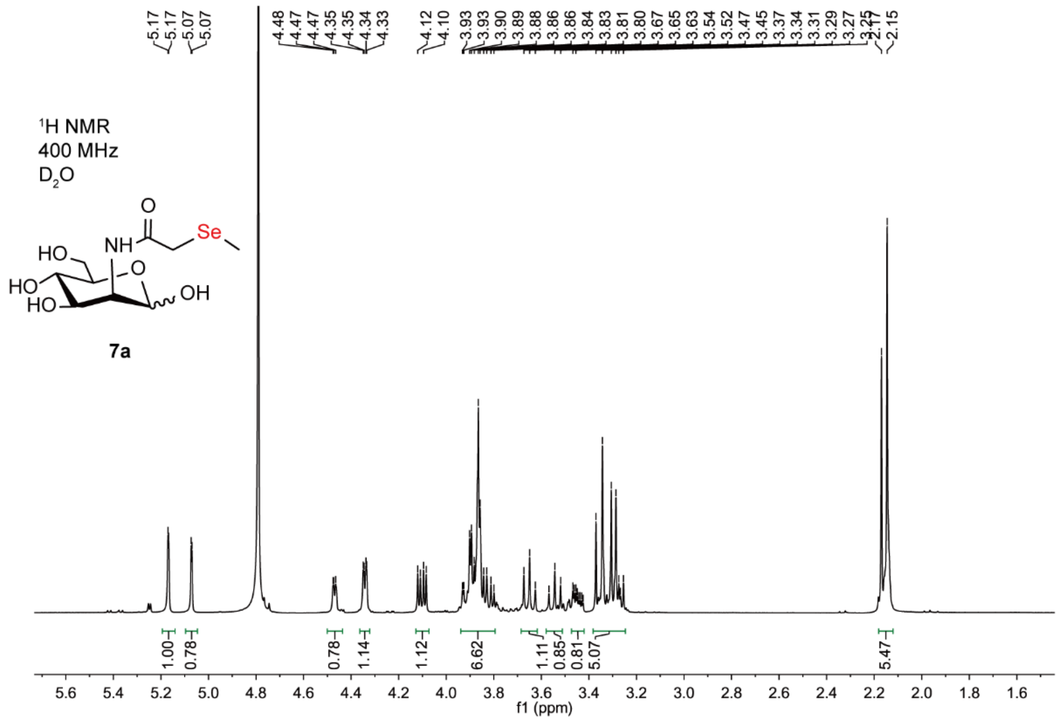

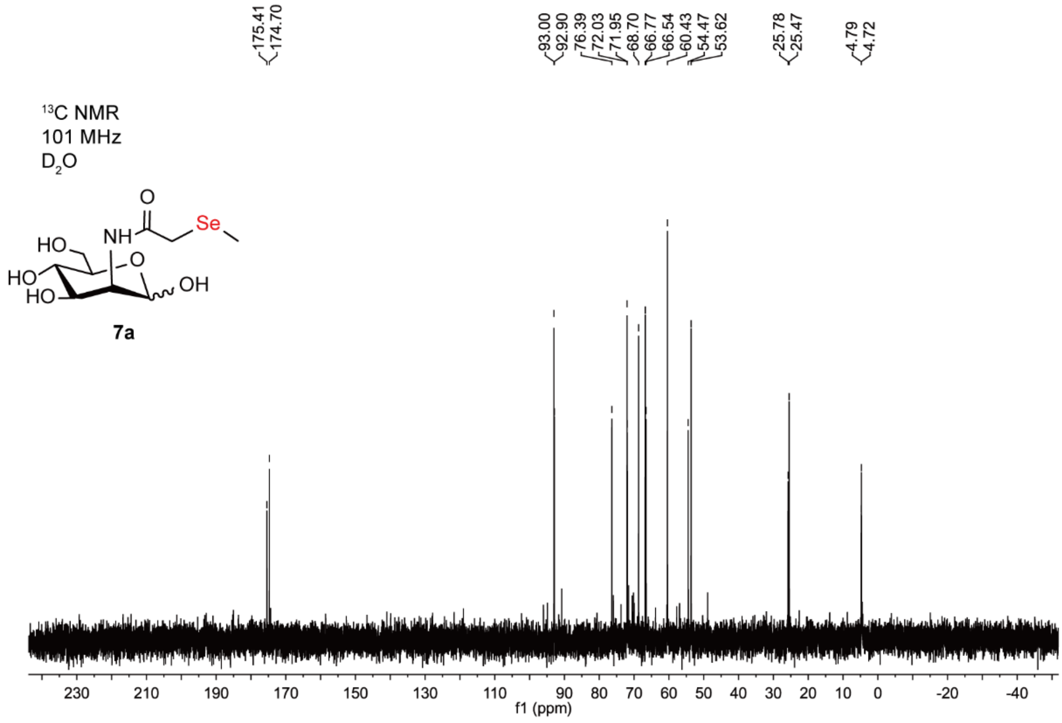

Supplementary Fig. 33 ^{1}H NMR and ^{13}C NMR spectrum of **7a**

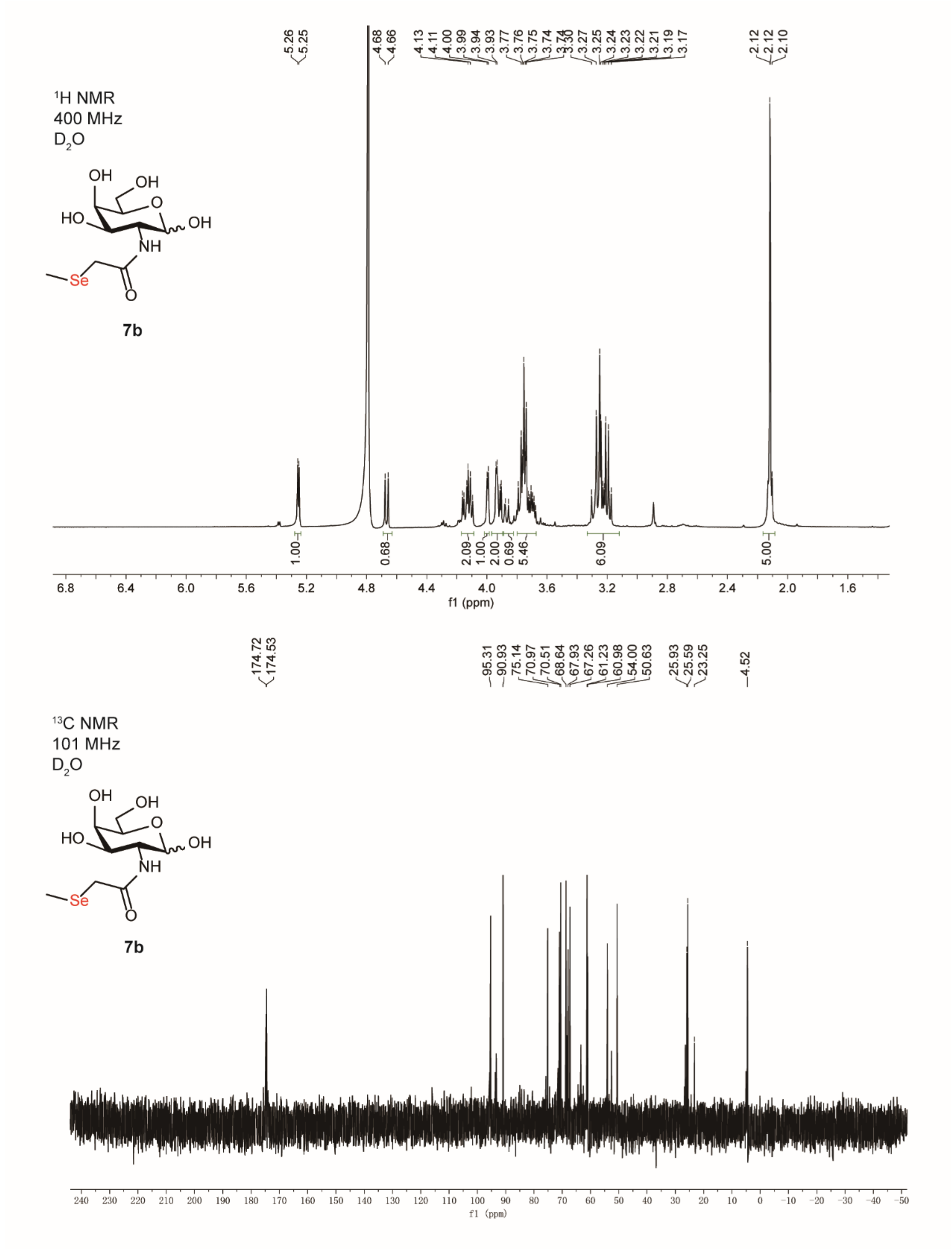

Supplementary Fig. 34 ^{1}H NMR and ^{13}C NMR spectrum of **7b**

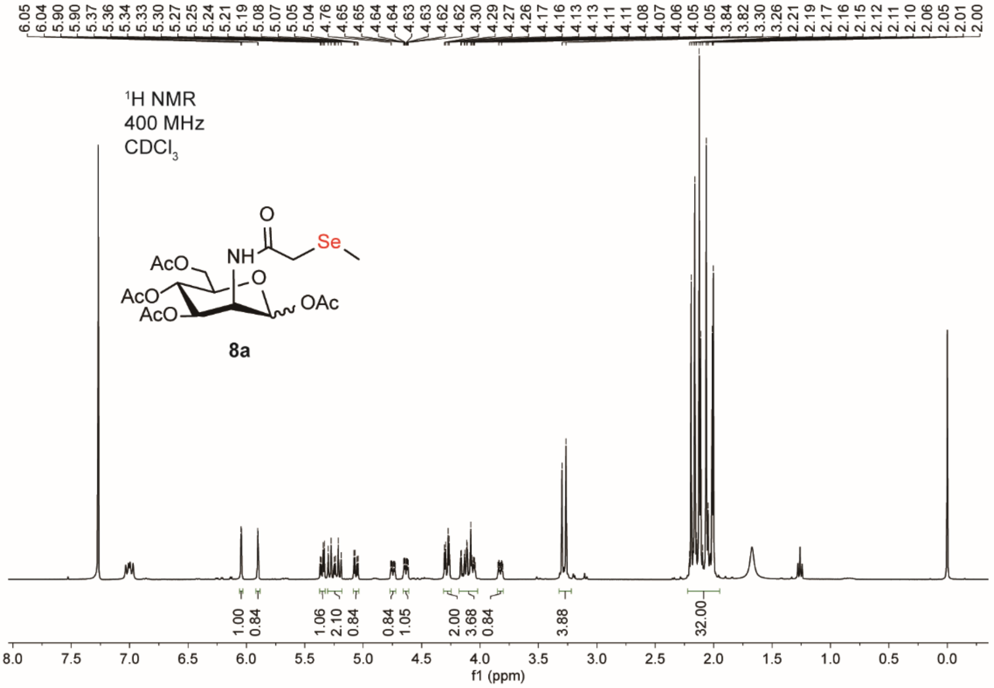

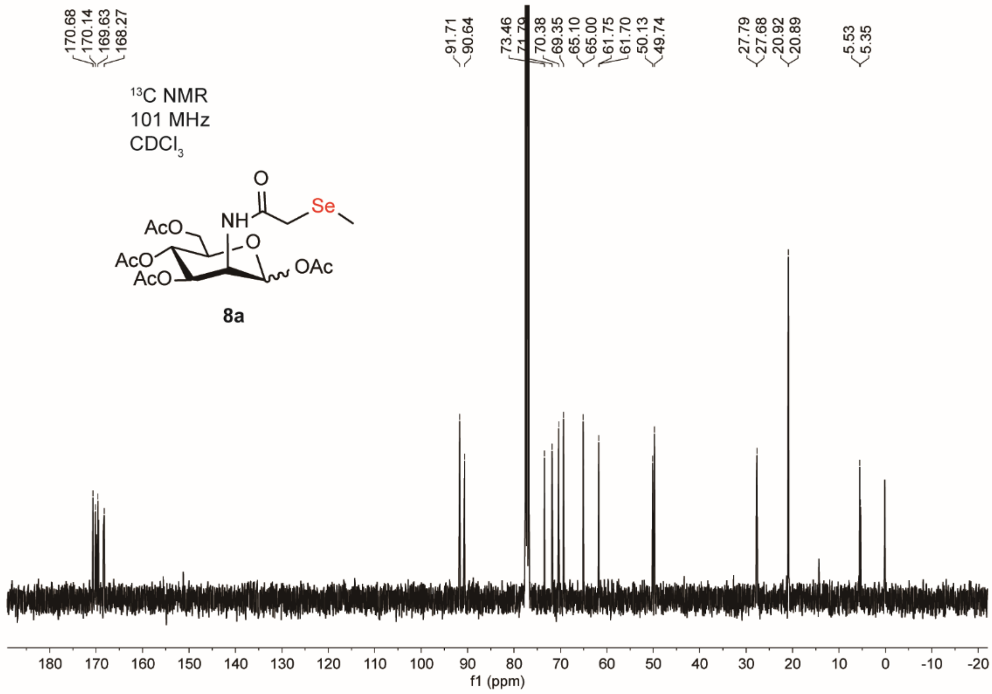

Supplementary Fig. 35 ^{1}H NMR and ^{13}C NMR spectrum of **8a**

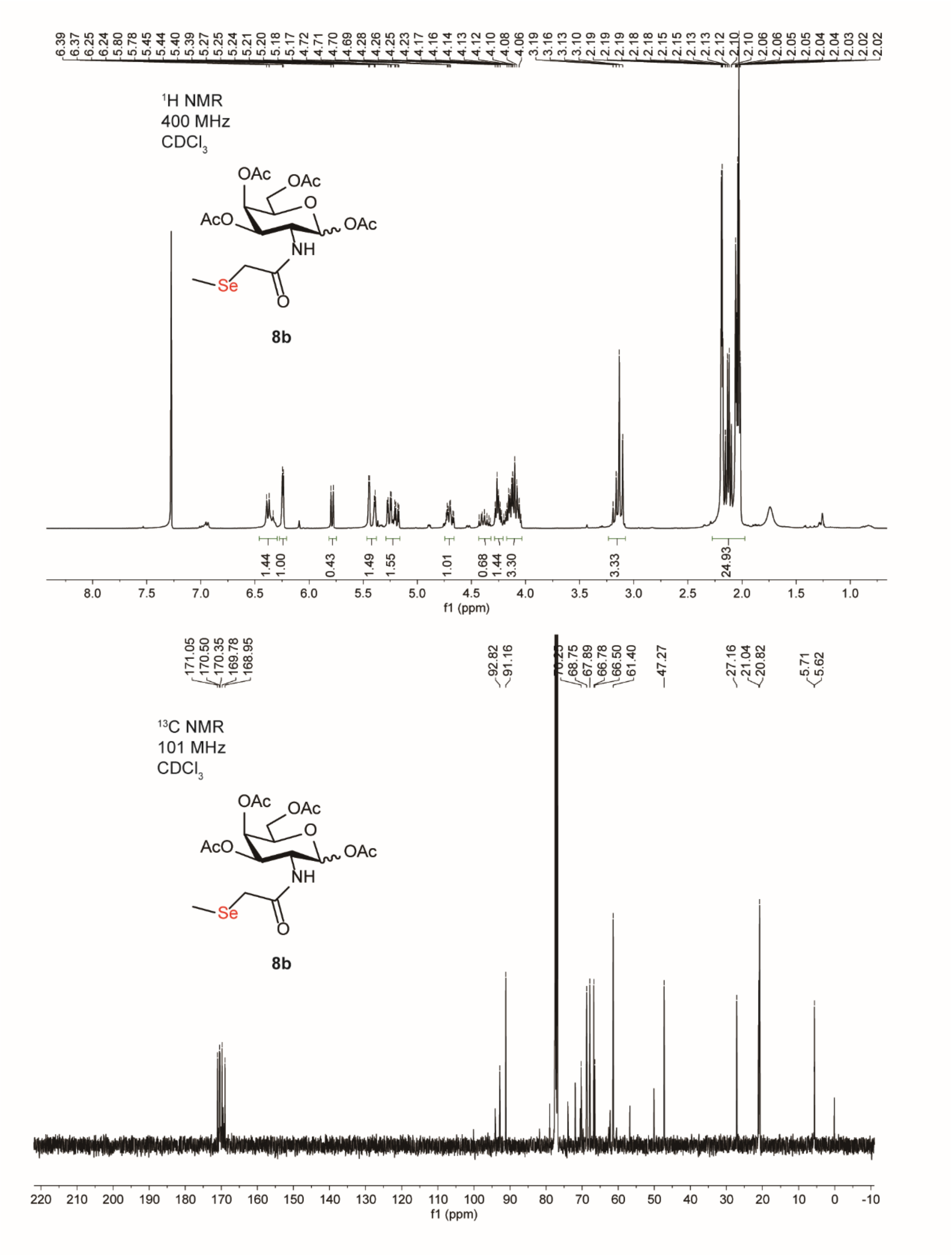

Supplementary Fig. 36 ^{1}H NMR and ^{13}C NMR spectrum of **8b**

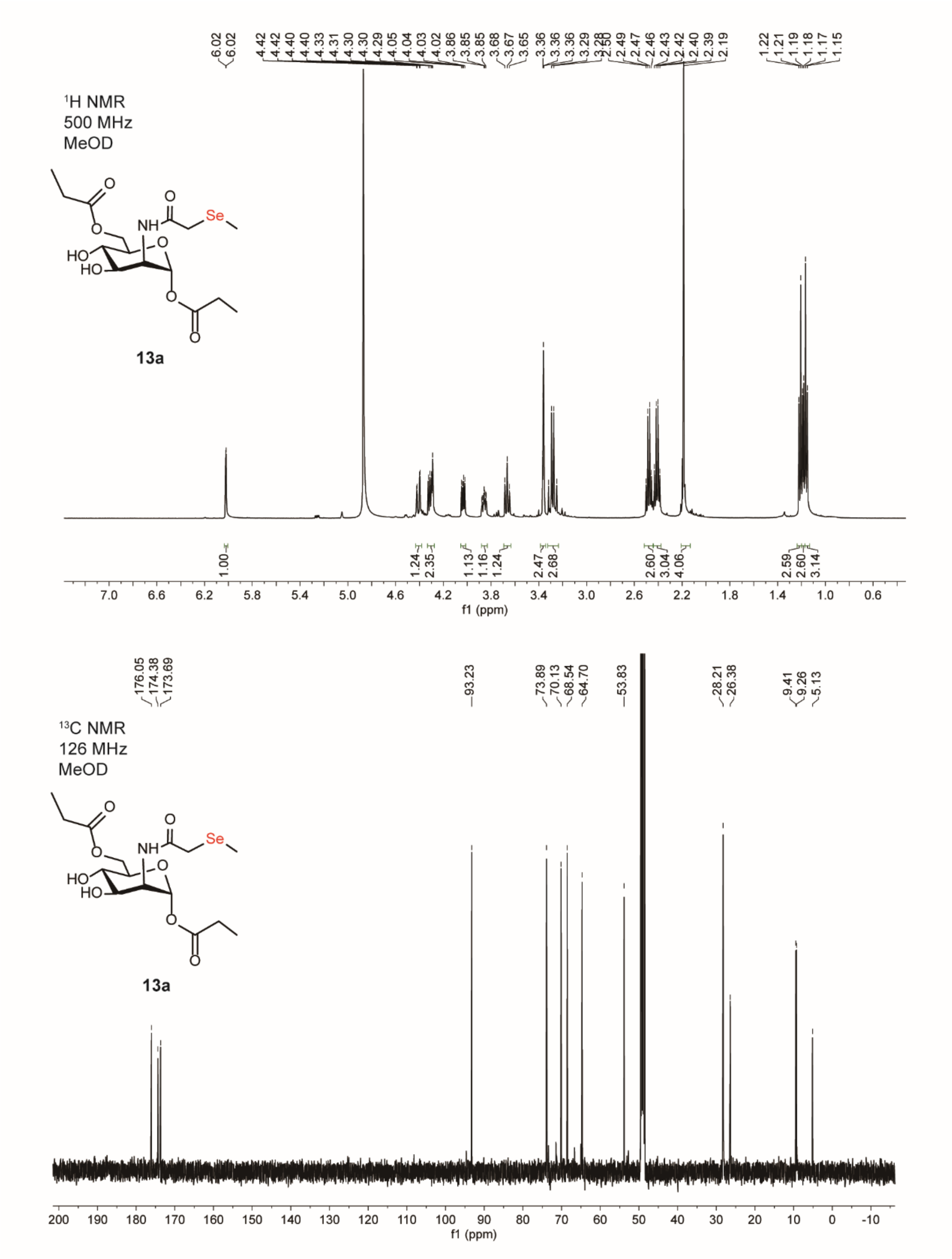

Supplementary Fig. 37 ^{1}H NMR and ^{13}C NMR spectrum of **13a**

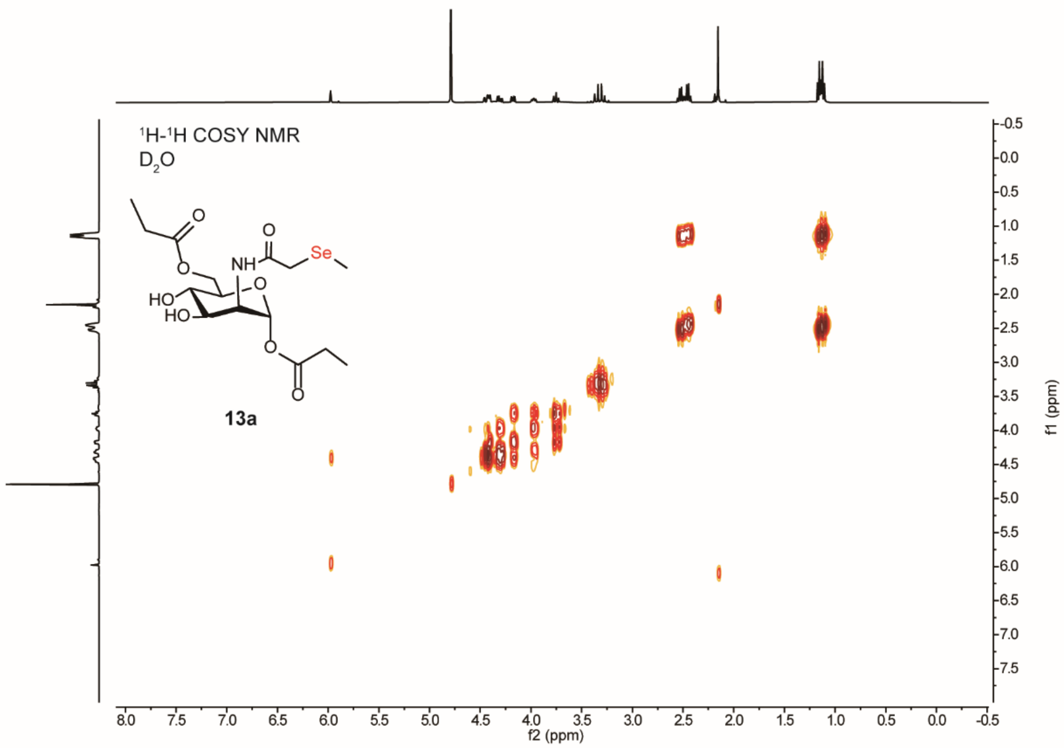

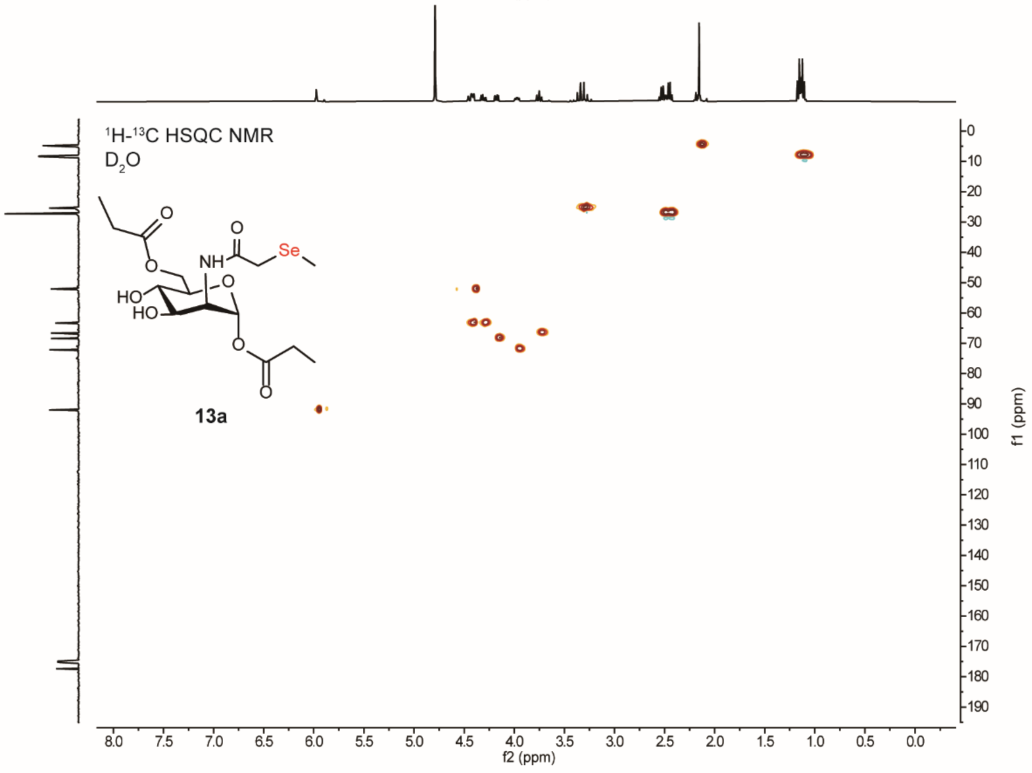

Supplementary Fig. 38 ^{1}H-^{1}H COSY NMR and ^{1}H-^{13}C HSQC NMR spectrum of **13a**

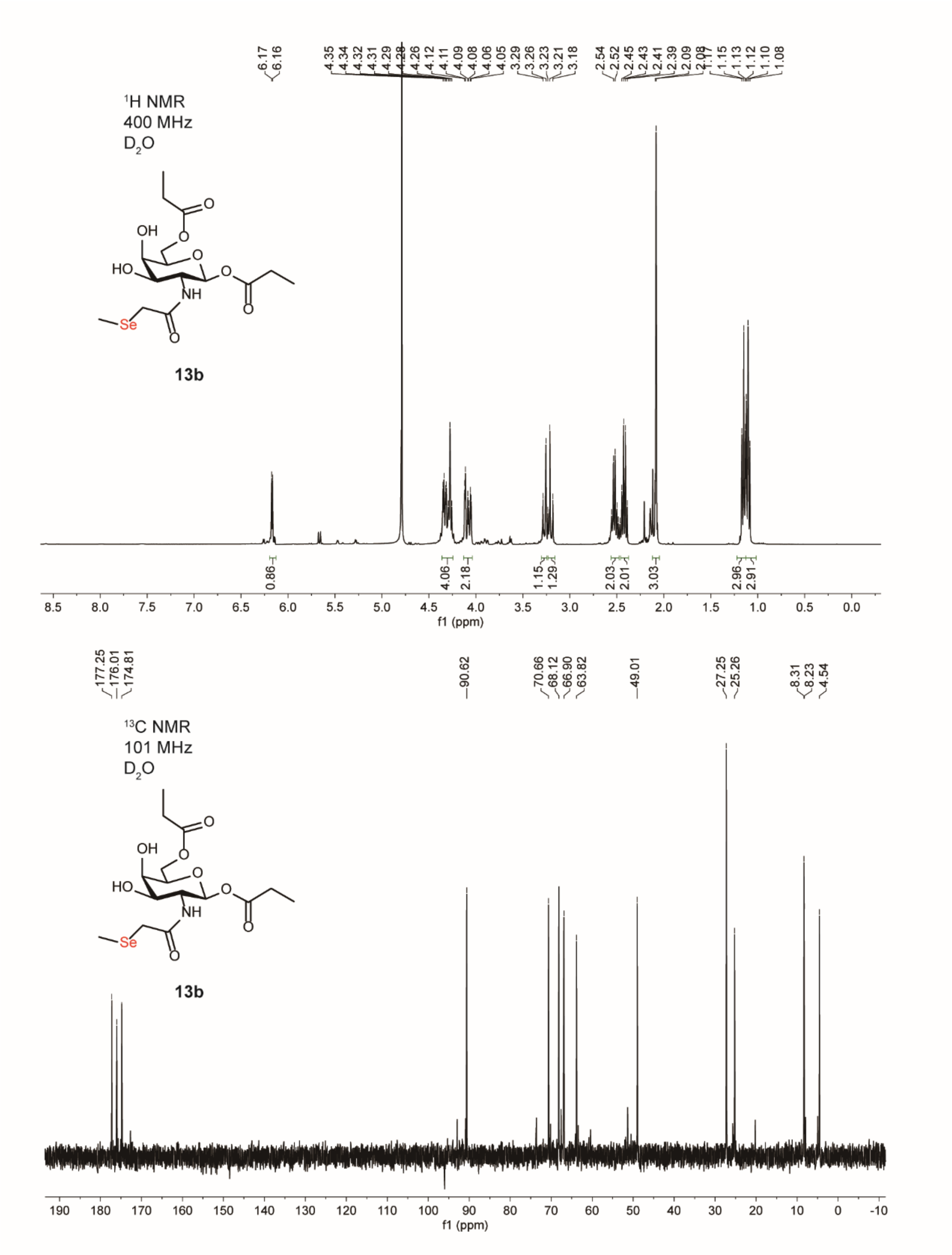

Supplementary Fig. 39 ^{1}H NMR and ^{13}C NMR spectrum of **13b**

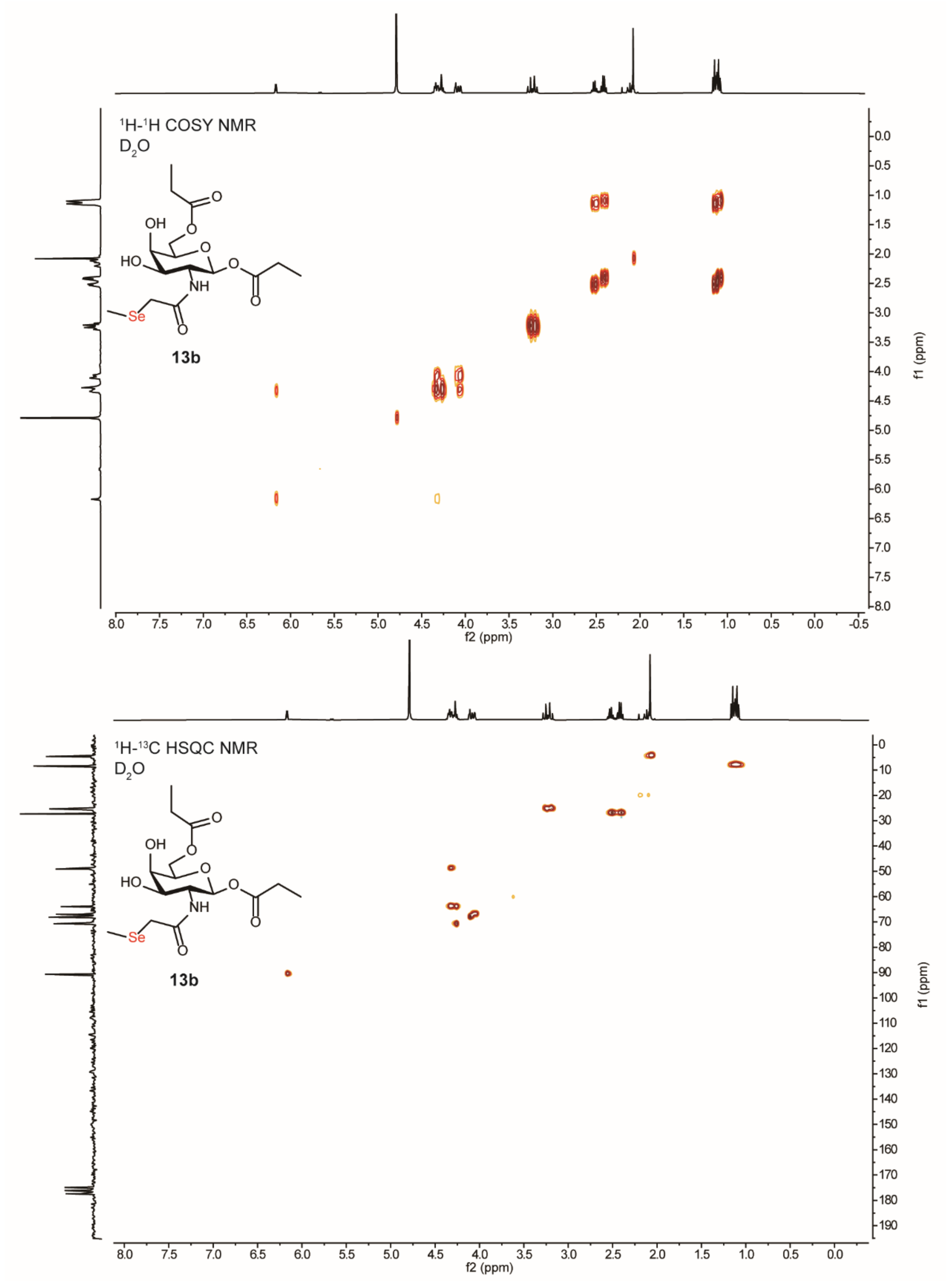

Supplementary Fig. 40 ^{1}H-^{1}H COSY NMR and ^{1}H-^{13}C HSQC NMR spectrum of **13b**

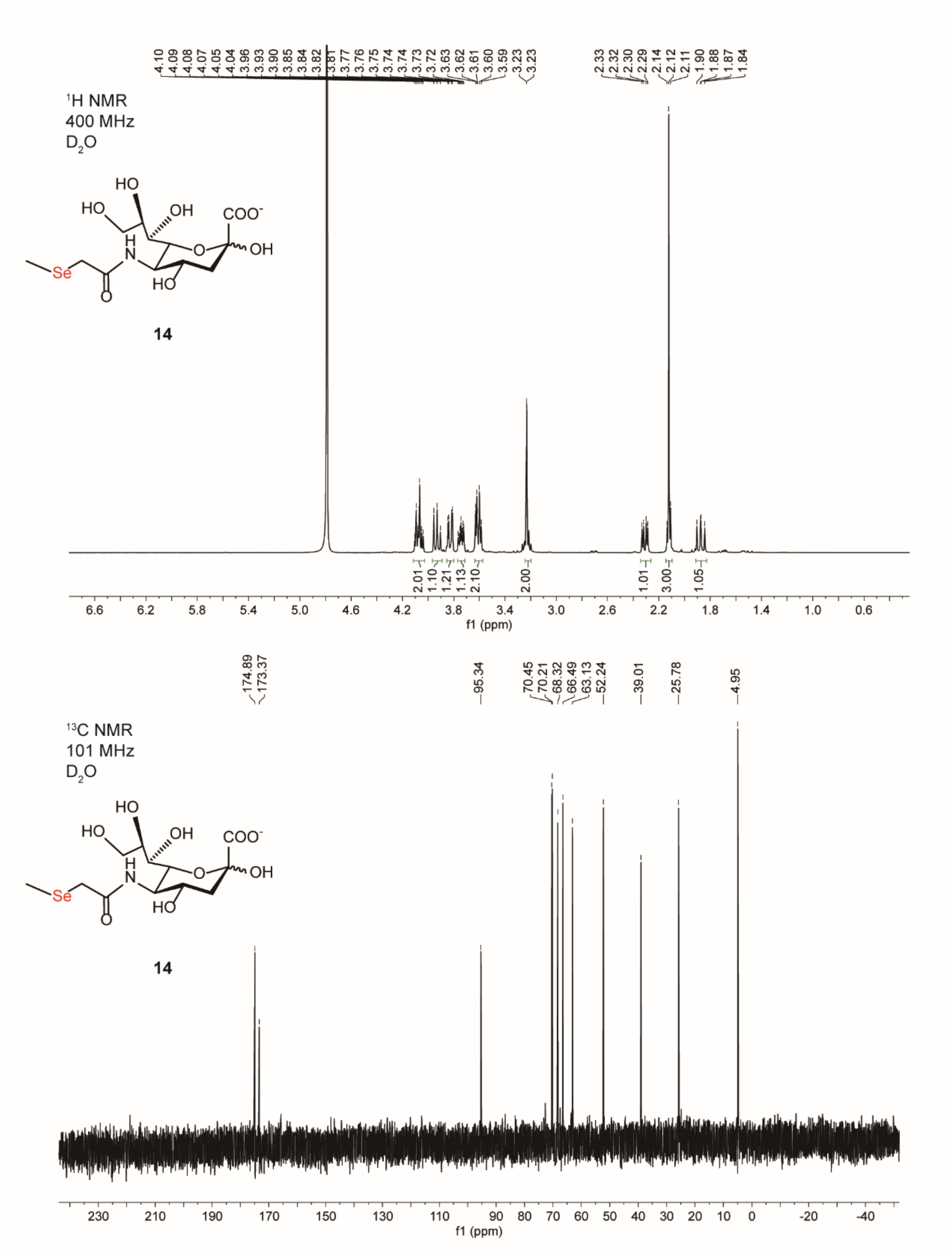

Supplementary Fig. 41 ^{1}H NMR and ^{13}C NMR spectrum of **14**

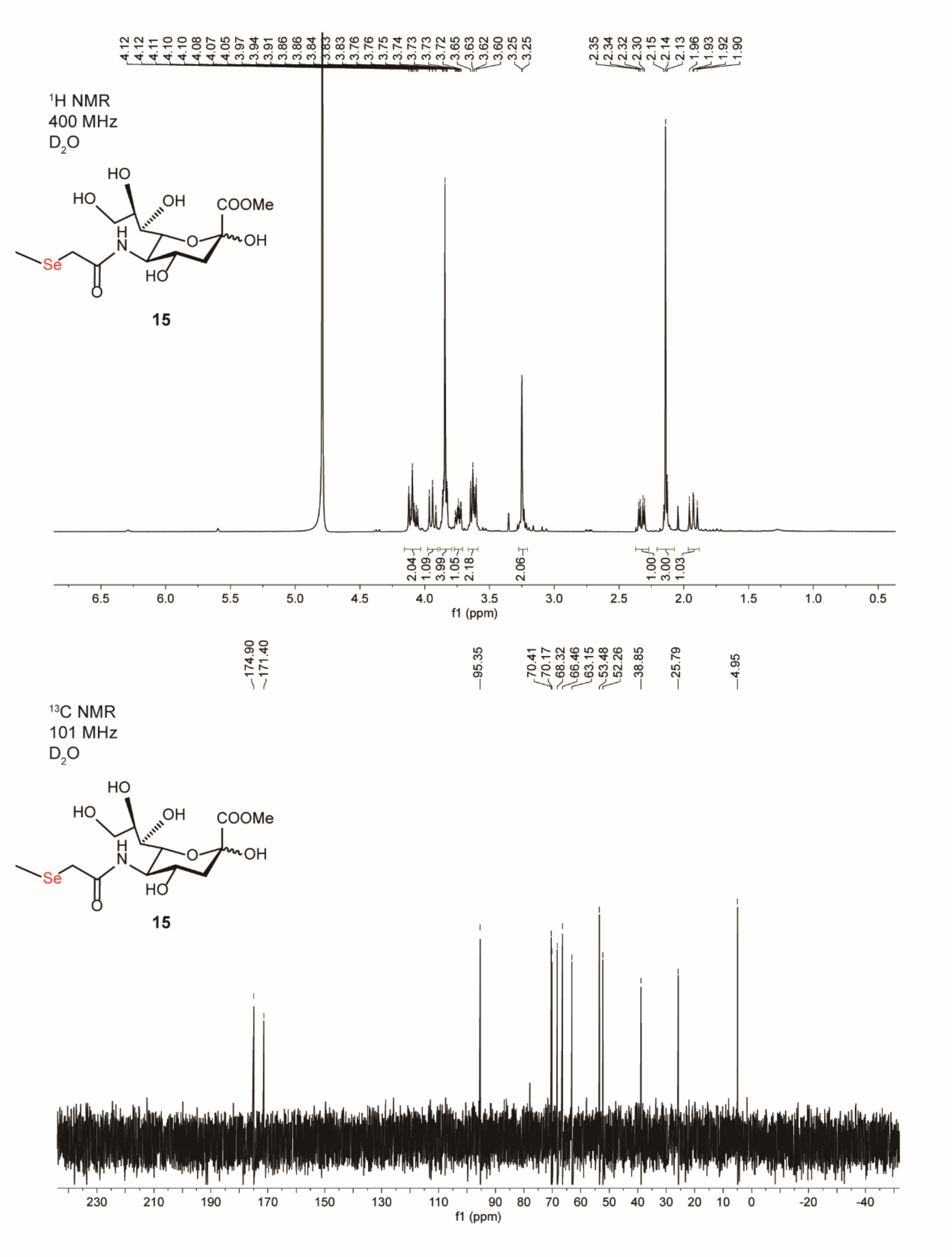

Supplementary Fig. 42 ^{1}H NMR and ^{13}C NMR spectrum of **15**

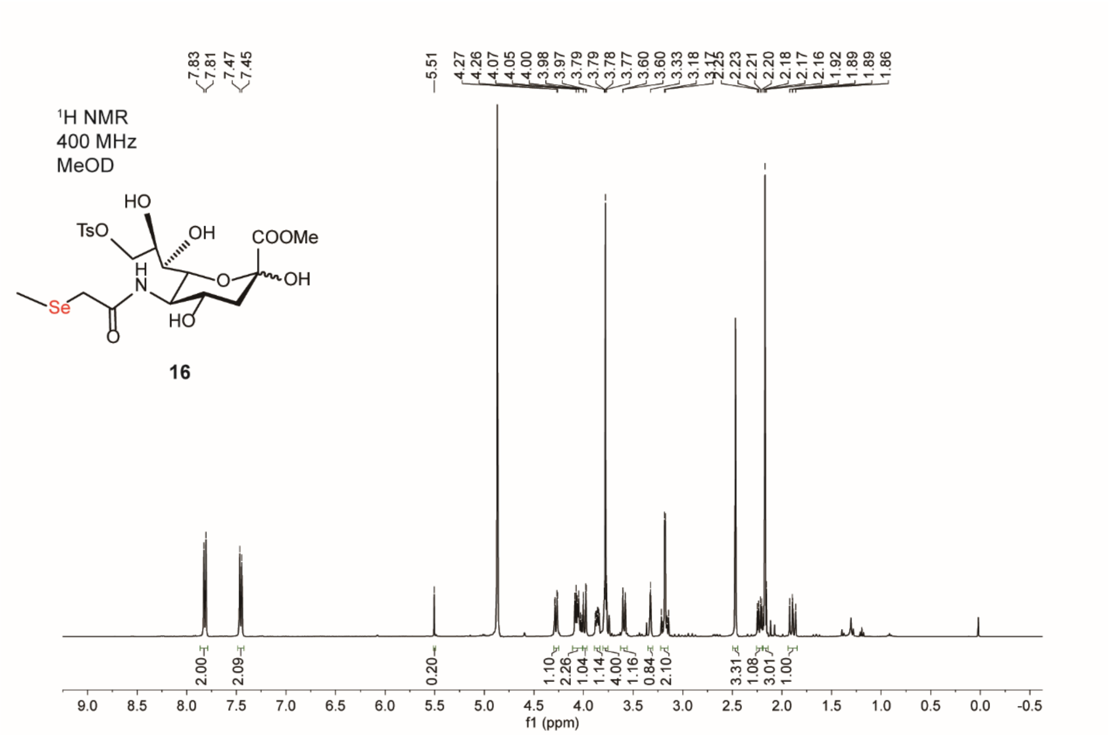

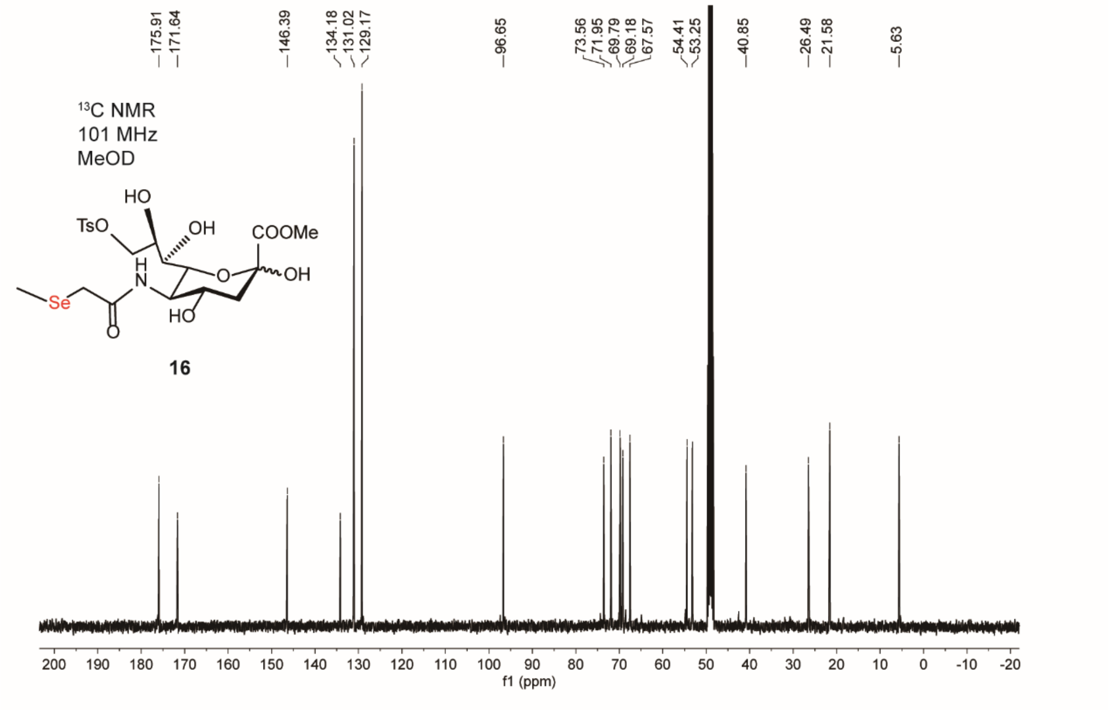

Supplementary Fig. 43 ^{1}H NMR and ^{13}C NMR spectrum of **16**

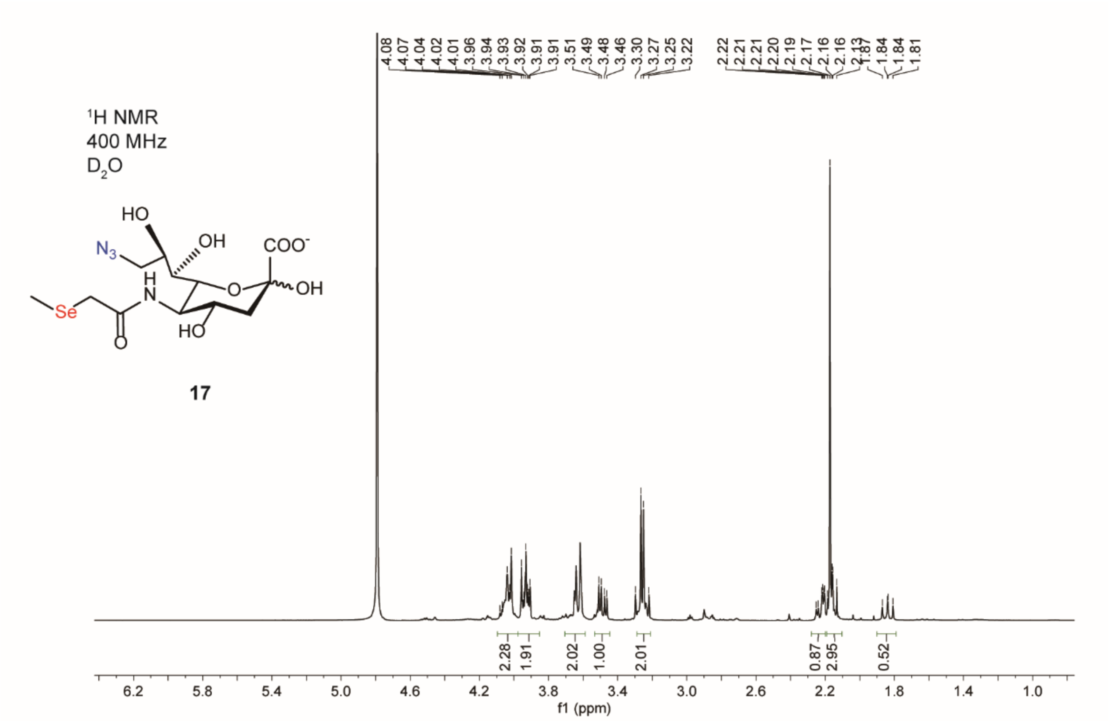

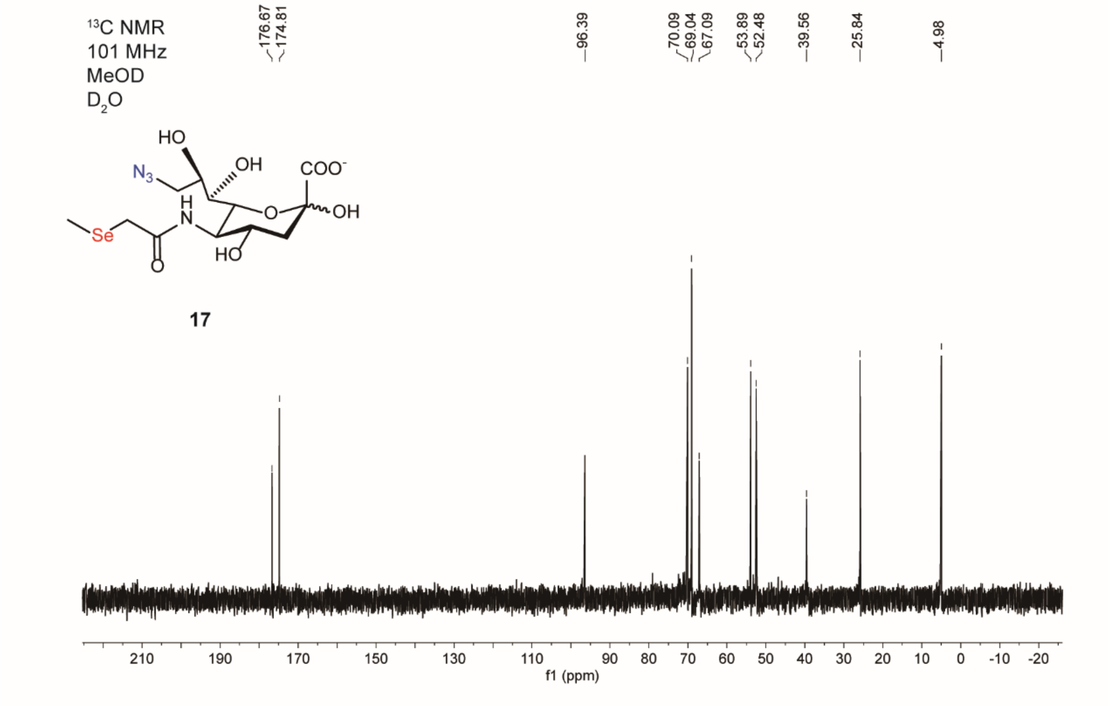

Supplementary Fig. 44 ^{1}H NMR and ^{13}C NMR spectrum of **17**

Supplementary Note 1: Synthetic procedures

General synthetic chemistry instrumentation

SiliCycle silica plates (TLG R10011B-624) were used for thin layer chromatography (TLC) with detection of multiband UV-absorption (254 to 365 nm). Column chromatography was carried out using SepaBean™ machine T flash chromatography system with an automated fraction collector. Semi-prep HPLC was carried out using a Waters LC Prep 150 system equipped with a 2998 photodiode array detector, an automatic fraction collector and a XBridge Prep C18 column (19 mm×150 mm, 5 µm) or a XBridge Prep amide column (19 mm×250 mm, 5 µm). Proton nuclear magnetic resonanc (^{1}H NMR) and proton-decoupled carbon-13 nuclear magnetic resonance (13C {1H} NMR) spectra were obtained on a 400 MHz Bruker AVANCE III-400 spectrometer at 25 °C. All chemical NMR analysis was conducted on MestReNova v12.0.3. Data are presented in the form of chemical shift, multiplicity, coupling constants in Hertz (Hz), and integration. Chemical shifts are reported in δ (ppm) relative to the solvent residual peak. Coupling constants are reported in Hz with multiplicities denoted as s (singlet), d (doublet), t (triplet), q (quartet), m (multiplet) and dd (doublet of doublet). High resolution mass spectrometry (HR-MS) was conducted on a Thermo Fisher Q Exactive LC/MS.

Synthesis of 2-(methylselanyl)acetic acid NHS ester

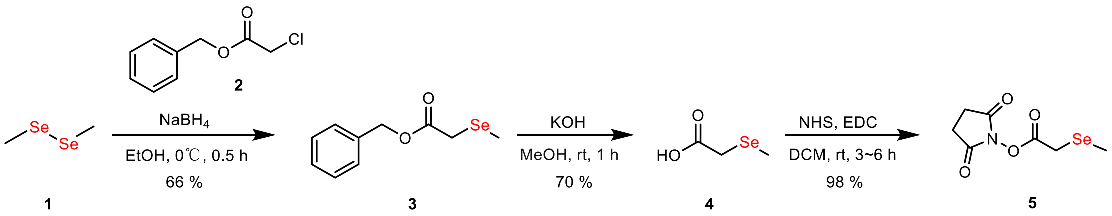

Benzyl 2-(methylselanyl)acetate (3): $NaBH_4$ (2.31 g, 61.18 mmol, 2.3 eq.) was added slowly to a stirred solution of **1** (5.0 g, 26.60 mmol, 1.0 eq.) in anhydrous EtOH (150 mL) at 0°C under a nitrogen atmosphere. The reaction mixture was stirred for ~10 min at 0°C until the characteristic diselenide yellow color disappeared. Subsequently, **2** (10.8 g, 58.52 mmol, 2.2 eq.) was added dropwise in 5 min, with the observation of immediate precipitation of a white solid, and the reaction mixture was then stirred for 0.5 h, and monitored using TLC. Deionized water (10 mL) and NaCl (5 g) was added to quench the reaction, the product was extracted with diethyl ether (4 x 150 mL), dried over Na_2SO_4, filtered, and concentrated to a yellow oil. Purification via silica gel flash column chromatography eluted with PE gave **3** as a yellow oil (8.54 g, 35.12 mmol, 66%). **TLC** (Rf = 0.3, PE: EA=30:1, stained by phosphomolybdic acid). **^{1}H NMR** (400 MHz, $CDCl_3$) *δ* 7.40 – 7.31 (m, 5H), 5.16 (s, 2H), 3.18 (s, 2H), 2.14 (s, 3H). **^{13}C NMR** (101 MHz, $CDCl_3$) δ 171.32, 135.83, 128.67, 128.41, 128.31, 66.98, 23.71, 6.01. **ESI-HRMS:** Calcd. for $C_{10}H_{12}O_2SeNa$ $[M+Na]^+$ = 266.9895, found 266.9870

2-(methylselanyl)acetic acid (4): To a solution of **3** (8.54 g, 35.12 mmol, 1.0 eq.) in $MeOH/H_2O$ (3/1; 80 mL) at 0°C, KOH (2.76 g, 49.17 mmol, 1.4 eq.) was added slowly. The reaction was warmed to room temperature and stirred for 1 h, diluted with deionized water (50

mL), and EtOAc (4 x 70 mL). The aqueous layer was collected, followed by addition of HCl (1 M aqueous solution) to pH 4. Then, the aqueous layer was extracted with CH_2Cl_2 (4 x 200 mL). The pooled organic extracts were dried over Na_2SO_4, filtered, and concentrated to obtain a colorless liquid (3.76 g, 24.58 mmol, 70%). **^{1}H NMR** (400 MHz, DMSO-*d*$_6$) δ 3.12 (s, 1H), 2.08 (s, 1H). **^{13}C NMR** (101 MHz, DMSO-*d*$_6$) δ 172.43, 23.88, 5.21. **ESI-HRMS**: Calcd. for $C_3H_5O_2Se^-$ [M-H]$^-$ = 152.9533, found 152.9440

2,5-dioxopyrrolidin-1-yl 2-(methylselanyl)acetate (5): To a solution of **4** (3.5 g, 22.87 mmol, 1.0 eq.) in CH_2Cl_2 (80 mL), *N*-hydroxysuccinimide (2.76 g, 24.01 mmol, 1.05 eq.) and 1-ethyl(3-dimethylaminopropyl)carbodiimide (EDC) (8.77 g, 45.74 mmol, 2.0 eq.) were added. The reaction was stirred at room temperature for 3-6 h, concentrated *in vacuo* to give **5** as a white solid (5.61 g, 22.41 mmol, 98 %). The product was used immediately for the next step without any purification. **TLC** (Rf = 0.5, PE: EA=1:1, stained by iodine).

Synthesis of ManNSe, GalNSe

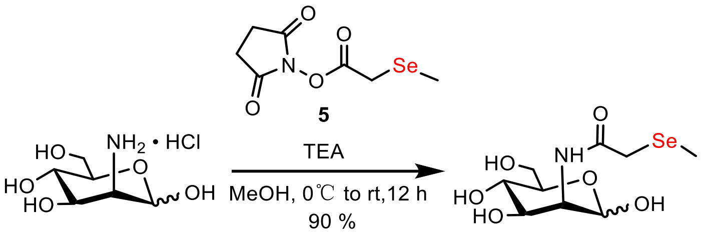

6a **7a**

2-(methylselanyl)-N-((*3S,4R,5S,6R*)-2,4,5-trihydroxy-6-(hydroxymethyl)tetrahydro-*2H*-pyran-3-yl)acetamide (7a, ManNSe): Under a nitrogen atmosphere, a solution of **6a** (3.0 g, 13.91 mmol, 1.0 eq.) and triethylamine (TEA) (6 mL, 41.73 mmol, 3.0 eq.) in anhydrous MeOH (100 mL) was cooled to 0°C, and then **5** (3.47 g, 13.91 mmol, 1.0 eq.) was added. The reaction was warmed to room temperature while stirred overnight, and concentrated *in vacuo* as a yellow oil. A first purification via silica gel flash column chromatography (5-20 % MeOH in DCM) gave **7a** as a light-yellow solid (3.93 g, 12.52 mmol, 90%). The crude product was further purified by normal phase HPLC (XBridge Prep amide column,19 mm×250 mm, 5 µm) to give the product as white solid, eluted with 0-95% water in ACN at the flow rate of 7 mL/min. **TLC** (Rf = 0.4, DCM:MeOH=3:1, stained by 10% H_2SO_4 in MeOH). **^{1}H NMR:** α: β=1:0.78 (400 MHz, D_2O) δ 5.17 (d, *J* = 1.0 Hz, 1H), 5.07 (d, *J* = 1.5 Hz, 1H), 4.44 (dd, *J* = 4.4, 1.4 Hz, 0.78H), 4.34 (dd, *J* = 4.6, 1.3 Hz, 1H), 4.10 (dd, *J* = 9.8, 4.7 Hz, 1H), 3.96 – 3.79 (m, 6H), 3.65 (t, *J* = 9.6 Hz, 1H), 3.54 (t, *J* = 9.8 Hz, 1H), 3.45 (ddd, *J* = 9.9, 5.1, 2.3 Hz, 1H), 3.38 – 3.25 (m, 5H), 2.16 (d, *J* = 9.6 Hz, 5H). **^{13}C NMR:** (anomers, 101 MHz, D_2O) δ 175.70, 174.41, 93.00, 92.90, 76.39, 72.03, 71.95, 68.70, 66.77, 66.54, 60.43, 54.47, 53.62, 25.78, 25.47, 4.79, 4.72. **ESI-HRMS**: Calcd. for $C_9H_{17}NO_6SeNa$ [M+Na]$^+$= 338.0114, found 338.0101

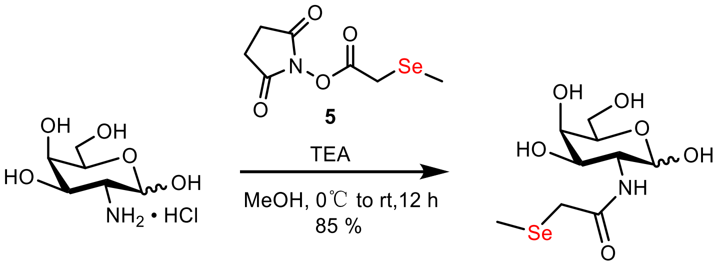

6b **7b**

2-(methylselanyl)-N-((*3R,4R,5R,6R*)-2,4,5-trihydroxy-6-(hydroxymethyl)tetrahydro-*2H*-pyran-3-yl)acetamide (7b, GalNSe): 7b was obtained with the same procedure for synthesis of **7a** as a white solid. **TLC** (Rf = 0.4, DCM: MeOH=3:1, stained by 10% H_2SO_4 in MeOH). **^{1}H NMR:** α: β=1:0.68 (400 MHz, D_2O) δ 5.25 (d, *J* = 3.7 Hz, 1H), 4.67 (d, *J* = 8.4 Hz, 0.68H), 4.18 – 4.08 (m, 2H), 3.99 (d, *J* = 3.0 Hz, 1H), 3.92 (dd, *J* = 11.2, 3.0 Hz, 2H), 3.89 – 3.84 (m, 0.68H), 3.80 – 3.66 (m, 5H), 3.32 – 3.15 (m, 6H), 2.20 – 2.03 (m, 5H). **^{13}C NMR**: (anomers, 101 MHz, D_2O) δ174.71, 174.53, 95.31, 90.92, 75.13, 70.96, 70.51, 68.64, 68.42, 67.92, 67.26, 61.23, 60.98, 54.00, 50.62, 25.92, 25.59, 4.52, 4.44. **ESI-HRMS**: Calcd. for $C_9H_{17}NO_6SeNa$ $[M+Na]^+$ = 338.0114, found 338.0102

Synthesis of Ac4ManNSe, Ac4GalNSe

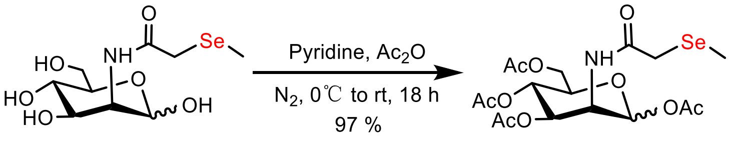

7a **8a**

(*3S,4R,5S,6R*)-6-(acetoxymethyl)-3-(2-(methylselanyl)acetamido)tetrahydro-*2H*-pyran-2,4,5-triyl triacetate (8a, Ac4ManNSe): 7a (1.0 g, 3.18 mmol) was dissolved in pyridine (50 mL), cooled to 0°C, and acetic anhydride (25 mL) was added. The reaction was warmed to room temperature and stirred for 18 h. The reaction mixture was diluted with ethyl acetate, and sequentially washed with 1 M HCl, sat. aq. $NaHCO_3$, and brine. The organic phase was dried over Na_2SO_4, filtered, and concentrated in vacuum. The residue was first purified by silica gel flash column chromatography (5-50% EA in PE). Further purification via reversed phase HPLC (0-90% ACN in water at the flow rate of 7 mL/min) gave **8a** as a white solid (1.49 g, 3.09 mmol, 97%). **TLC** (Rf = 0.5, PE: EA =1:1, stained by 10% H_2SO_4 in MeOH). **^{1}H NMR**: α: β=1:0.84 (400 MHz, $CDCl_3$) δ 6.04 (d, *J* = 1.7 Hz, 1H), 5.90 (d, *J* = 1.6 Hz, 0.84H), 5.35 (dd, *J* = 10.3, 4.1 Hz, 1H), 5.31 – 5.18 (m, 2H), 5.06 (dd, *J* = 10.0, 3.8 Hz, 0.84H), 4.75 (ddd, *J* = 9.3, 3.8, 1.5 Hz, 0.84H), 4.63 (ddd, *J* = 9.7, 4.0, 1.9 Hz, 1H), 4.28 (dd, *J* = 12.4, 3.9 Hz, 2H), 4.17 – 4.03 (m, 4H), 3.83 (ddd, *J* = 9.8, 4.3, 2.4 Hz, 0.84H), 3.28 (d, *J* = 14.0 Hz, 4H), 2.22 – 1.99 (m, 32H). **^{13}C NMR**: (anomers, 101 MHz, $CDCl_3$) δ 170.68, 170.14, 169.63, 168.27, 91.71, 90.64, 73.46, 71.79, 70.38, 69.35, 65.10, 65.00, 61.75, 61.70, 50.13, 49.74, 27.79, 27.68, 20.92, 20.89, 5.53, 5.35. **ESI-HRMS**: Calcd. for $C_{17}H_{25}NO_{10}SeNa$ $[M+Na]^+$ = 506.0536, found 506.0527

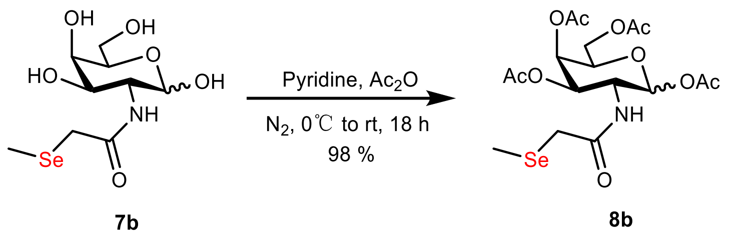

(*3R,4R,5R,6R*)-6-(acetoxymethyl)-3-(2-(methylselanyl)acetamido)tetrahydro-*2H*-pyran-2,4,5-triyl triacetate (8b, Ac_4GalNSe): **8b** was obtained from **7b** with the same procedure for synthesis of **8a** as a white solid. **TLC** (Rf = 0.5, PE:EA =1:1, stained by 10% H_2SO_4 in MeOH). **^{1}H NMR**: α: β=1:0.43 (400 MHz, $CDCl_3$) δ 6.46 – 6.29 (m, 1.44H), 6.24 (d, *J* = 3.5 Hz, 1H), 5.79 (d, *J* = 8.8 Hz, 0.43H), 5.42 (dd, *J* = 21.3, 3.2 Hz, 1.49H), 5.29 – 5.16 (m, 1.55H), 4.74 – 4.65 (m, 1H), 4.43 – 4.32 (m, 0.68H), 4.25 (dt, *J* = 10.9, 5.5 Hz, 1.44H), 4.17 – 4.03 (m, 3H), 3.15 (dd, *J* = 23.5, 11.9 Hz, 3H), 2.27 – 1.98 (m, 25H). **^{13}C NMR**: (anomers, 101 MHz, $CDCl_3$) δ 171.05, 170.50, 170.35, 169.78, 168.95, 92.82, 91.16, 70.25, 68.75, 67.89, 66.78, 66.50, 61.40, 47.27, 27.16, 21.04, 20.82, 5.71, 5.62. **ESI-HRMS**: Calcd. for $C_{17}H_{25}NO_{10}SeNa$ $[M+Na]^+$= 506.0536, found 506.0527

Synthesis of 1,6-Pr_2ManNSe, 1,6-Pr_2GalNSe

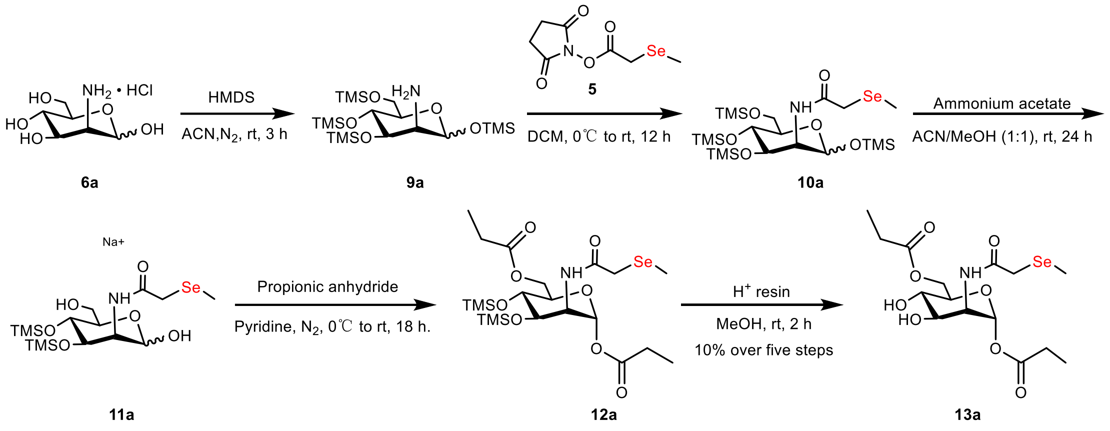

(*3S,4R,5R,6R*)-2,4,5-tris((trimethylsilyl)oxy)-6-(((trimethylsilyl)oxy)methyl)tetrahydro-*2H*-pyran-3-amine (9a): Under a nitrogen atmosphere, to a suspension of **1a** (*D*-Mannosamine·HCl, 5.0 g, 23.19 mmol, 1.0 eq.) in anhydrous ACN (100 mL), hexamethyldisilazane (HMDS) (12.3 mL, 57.97 mmol, 2.5 eq.) was added dropwise. The reaction was stirred at room temperature for 3 h, filtered to remove white precipitates, and the residue was concentrated to a colorless oil. The product was used immediately for the next step without further purification. **TLC** (Rf = 0.6, PE:EA =3:1, stained by 10% H_2SO_4 in MeOH)

2-(methylselanyl)-N-((*3S,4R,5R,6R*)-2,4,5-tris((trimethylsilyl)oxy)-6 (((trimethylsilyl)oxy)methyl)tetrahydro-*2H*-pyran-3-yl)acetamide (10a): A solution of **5** (5.8 g, 23.19 mmol, 1.0 eq) was cooled to 0°C and added to freshly-prepared **9a**. The reaction was warmed to room temperature and stirred overnight. The mixture was diluted with DCM, and washed with sat. aq. $NaHCO_3$. The organic phase was collected, dried over Na_2SO_4 and

concentrated to a yellow oil. The product was used immediately for the next step without further purification. **TLC** (Rf = 0.5, PE:EA =8:1, stained by 10% H_2SO_4 in MeOH)

N-((*3S,4R,5R,6R*)-2-hydroxy-6-(hydroxymethyl)-4,5-bis((trimethylsilyl)oxy)tetrahydro-*2H*-pyran-3-yl)-2-(methylselanyl)acetamide (11a): 10a obtained in the previous step was re-dissolved in the mixed solvent of ACN/MeOH (100 mL, 1:1 v/v), ammonia acetate (3.58 g, 46.38 mmol, 2.0 eq) was added. The reaction was stirred at room temperature for 24 h, concentrated *in vacuo*, diluted with EtOAc and washed four times with brine. The organic phase was collected, dried over Na_2SO_4, and concentrated to **11a** as a light-yellow oil. The product was used immediately for the next step without further purification. **TLC** (Rf = 0.6, DCM:MeOH =9:1, stained by 10% H_2SO_4 in MeOH). **ESI-HRMS**: Calcd. for $C_{15}H_{33}NO_6SeSi_2Na$ $[M+Na]^+$= 482.0904, found 482.0897

(*2R,3S,4R,5R,6R*)-3-(2-(methylselanyl)acetamido)-6-((propionyloxy)methyl)-4,5-bis((trimethylsilyl)oxy)tetrahydro-*2H*-pyran-2-yl propionate (12a)： 11a obtained in the previous step was re-dissolved in pyridine (80 mL), cooled to 0°C, and then propionic anhydride (45 mL) was added. The reaction was warmed to room temperature and stirred for 18 h. The reaction mixture was diluted sequentially with ethyl acetate, washed with 1 M HCl, sat. aq. $NaHCO_3$, and brine. The organic phase was dried over Na_2SO_4, filtered, and concentrated to a yellow oil. The product was used immediately for the next step without further purification. **TLC** (Rf = 0.6, PE:EA =3:1, stained by 10% H_2SO_4 in MeOH). **ESI-HRMS**: Calcd. for $C_{12}H_{41}NO_8SeSi_2Na$ $[M+Na]^+$= 574.1429, found 594.1436

(*2R,3S,4R,5S,6R*)-4,5-dihydroxy-3-(2-(methylselanyl)acetamido)-6-((propionyloxy)methyl)tetrahydro-*2H*-pyran-2-yl propionate (13a, 1,6-Pr_2ManNSe): 12a obtained in the previous step was re-dissolved in MeOH (100 mL), and then Dowex H^+ resin was added. The reaction was stirred at room temperature for 2 h. The mixture was filtrated and concentrated in vacuum. The residue was first purified by silica gel flash column chromatography (0-2% MeOH in DCM). Then further purification via reversed phase HPLC (10-100% ACN in water at the flow rate of 7 mL/min) gave **13a** as a white solid (1.03 g, 2.42 mmol). Total yield:10% over five steps. **TLC** (Rf = 0.5, DCM:MeOH =10:1, stained by 10% H_2SO_4 in MeOH). **^{1}H NMR**: (500 MHz, MeOD) δ 6.02 (d, *J* = 1.7 Hz, 1H), 4.41 (dd, *J* = 11.9, 2.2 Hz, 1H), 4.33 – 4.28 (m, 2H), 4.03 (dd, *J* = 9.4, 4.9 Hz, 1H), 3.88 – 3.83 (m, 1H), 3.67 (t, *J* = 9.7 Hz, 1H), 3.36 (dt, *J* = 3.3, 1.6 Hz, 2H), 3.28 (q, *J* = 12.5 Hz, 3H), 2.48 (q, *J* = 7.5 Hz, 3H), 2.41 (dd, *J* = 15.1, 7.6 Hz, 3H), 2.19 (s, 4H), 1.21 (d, *J* = 7.5 Hz, 3H), 1.19 (d, *J* = 4.8 Hz, 3H), 1.16 (d, *J* = 7.6 Hz, 3H). **^{13}C NMR**: (126 MHz, MeOD) δ 176.05, 174.38, 173.69, 93.23, 73.89, 70.13, 68.54, 64.70, 53.83, 28.21, 26.38, 9.41, 9.26, 5.13. **ESI-HRMS**: Calcd. for $C_{15}H_{25}NO_8SeNa$ $[M+Na]^+$= 450.0638, found 450.0620

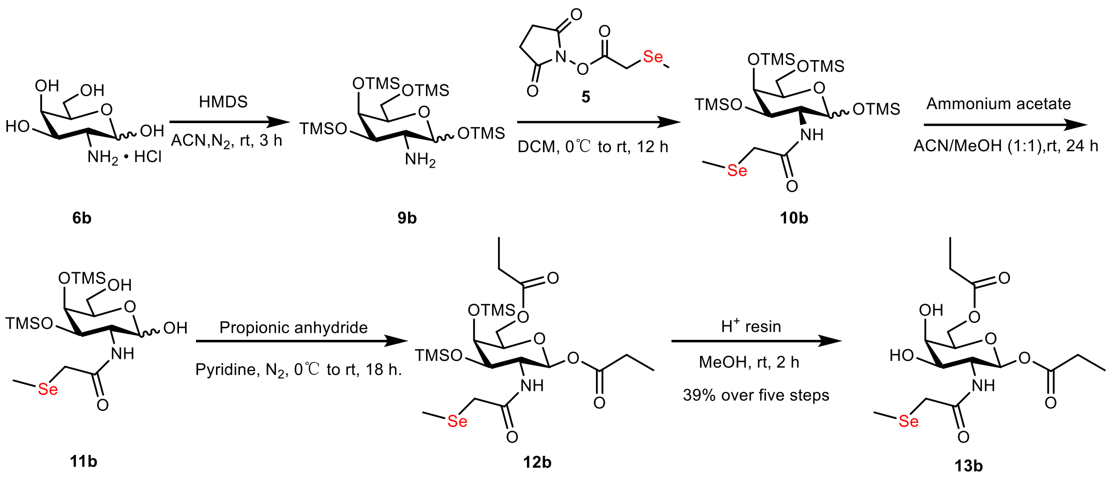

(*2S,3R,4R,5R,6R*)-4,5-dihydroxy-3-(2-(methylselanyl)acetamido)-6-((propionyloxy)methyl)tetrahydro-*2H*-pyran-2-yl propionate (13b, 1,6-Pr_2GalNSe): 13b (3.9 g,9.14 mmol, 1.0 eq.) was synthesized from **6b** following the procedure for the synthesis of **13a**. The total yield of **13b** was 39% over five steps. **TLC** (Rf = 0.5, DCM: MeOH =10:1, stained by 10% H_2SO_4 in MeOH). **^{1}H NMR**: (400 MHz, D_2O) δ 6.17 (d, *J* = 3.8 Hz, 1H), 4.40 – 4.23 (m, 4H), 4.14 – 4.04 (m, 2H), 3.27 (d, *J* = 12.6 Hz, 1H), 3.21 (t, *J* = 10.6 Hz, 1H), 2.57 – 2.47 (m, 2H), 2.47 – 2.38 (m, 2H), 2.09 (d, *J* = 4.3 Hz, 2H), 1.15 (t, *J* = 7.5 Hz, 3H), 1.10 (t, *J* = 7.6 Hz, 3H). **^{13}C NMR**: (101 MHz, D_2O) δ 177.25, 176.01, 174.81, 90.62, 70.66, 68.12, 66.90, 63.82, 49.01, 27.25, 25.26, 8.31, 8.23, 4.54. **ESI-HRMS**: Calcd. for $C_{15}H_{25}NO_8SeNa$ $[M+Na]^+$= 450.0638, found 450.0620

Synthesis of SiaNSe

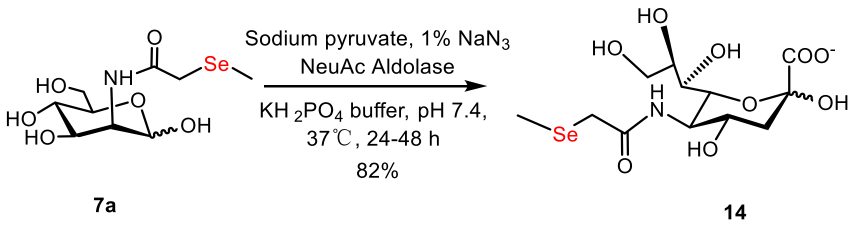

(*4S,5R*)-2,4-dihydroxy-5-(2-(methylselanyl)acetamido)-6-((*1R,2R*)-1,2,3-trihydroxypropyl)tetrahydro-*2H*-pyran-2-carboxylic acid (14, SiaNSe): 7a (1.0 g, 3.18 mmol, 1.0 eq.) was dissolved in 40 mL of 0.050 M potassium phosphate buffer (pH 7.4), followed by addition of sodium pyruvate (3.5 g, 31.8 mmol, 10.0 eq.), NaN_3 (final concentration of 1% w/v) and NeuAc aldolase (60-80 U). The mixture was reacted at 37°C for 24-48 h, concentrated *in vacuo*, and purified by anion-exchange chromatography with AG1X2 resin, formate form (Bio-Rad). The product was eluted with a gradient of 0.5 M to 1.0 M formic acid at 1.0 mL/min. The fractions containing the desired product were combined and concentrated in vacuum. Further purification via normal phase HPLC (XBridge Prep amide column,19 mm×250 mm, 5 µm) gave **14** as a white solid (1.0 g, 2.61 mmol, 82%), eluted with 0-95% water in ACN at the flow rate of 7 mL/min. **TLC** (Rf = 0.3, DCM:MeOH:H_2O = 3:2:0.5, stained by 10% H_2SO_4 in MeOH). **^{1}H NMR:** (400 MHz, D_2O) δ 4.12 – 4.03 (m, 2H), 3.97 – 3.89 (m, 1H), 3.83 (dd, *J* = 11.9, 2.6 Hz, 1H), 3.74 (ddd, *J* = 9.0, 6.2, 2.6 Hz, 1H), 3.64 – 3.57 (m, 2H),

3.23 (d, *J* = 1.1 Hz, 2H), 2.31 (dd, *J* = 13.0, 4.9 Hz, 1H), 2.15 – 2.09 (m, 3H), 1.87 (dd, *J* = 13.0, 11.5 Hz, 1H).**^{13}C NMR:** (101 MHz, D_2O) δ 174.89, 173.37, 95.34, 70.45, 70.21, 68.32, 66.49, 63.13, 52.24, 39.01, 25.78, 4.95. **ESI-HRMS**: Calcd. for $C_{12}H_{20}NO_9Se^-$ $[M-H]^-$= 402.0309, found 402.0302

Synthesis of 9AzSiaNSe

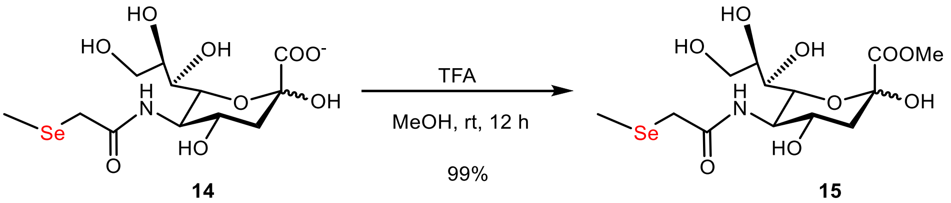

methyl (*4S,5R*)-2,4-dihydroxy-5-(2-(methylselanyl)acetamido)-6-((*1R,2R*)-1,2,3-trihydroxypropyl)tetrahydro-*2H*-pyran-2-carboxylate (15): To a solution of **14** (500 mg, 1.25 mmol,1.0 eq.) in 50 mL MeOH, 0.5 mL trifluoroacetic acid (TFA) was added. The reaction was stirred at room temperature overnight. The solvent was removed in vacuo, and the residue was purified by silica gel flash chromatography eluted with DCM: MeOH gradually from 15:1 to 9:1 to give the desired product as a white solid (515 mg, 1.24 mmol, 99%). **TLC** (Rf = 0.4, DCM: MeOH =4:1, stained by 10% H_2SO_4 in MeOH). **^{1}H NMR:** (400 MHz, D_2O) δ 4.09 (ddd, *J* = 16.8, 9.1, 2.9 Hz, 2H), 3.94 (t, *J* = 10.3 Hz, 1H), 3.88 – 3.79 (m, 4H), 3.77 – 3.70 (m, 1H), 3.62 (dd, *J* = 11.2, 6.3 Hz, 2H), 3.25 (d, *J* = 0.9 Hz, 2H), 2.33 (dd, *J* = 13.1, 4.9 Hz, 1H), 2.21 – 2.07 (m, 3H), 1.93 (dd, *J* = 13.1, 11.5 Hz, 1H).**^{13}C NMR:** (101 MHz, D_2O) δ 174.90, 171.40, 95.35, 70.41, 70.17, 68.32, 66.46, 63.15, 53.48, 52.26, 38.85, 25.79, 4.95. **ESI-HRMS:** Calcd. for $C_{13}H_{23}NO_9SeNa$ $[M+Na]^+$= 440.0431, found 440.0421

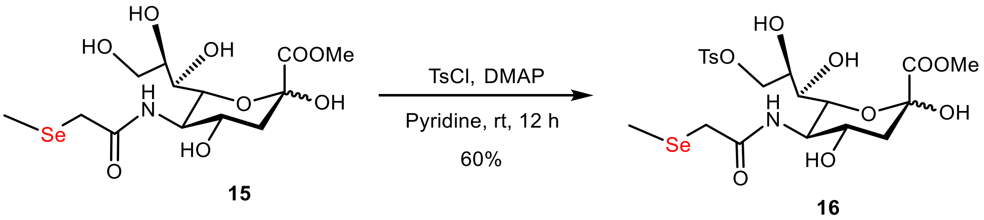

Methyl (*4S,5R*)-6-((*1R,2R*)-1,2-dihydroxy-3-(tosyloxy)propyl)-2,4-dihydroxy-5-(2-(methylselanyl)acetamido)tetrahydro-*2H*-pyran-2-carboxylate (16): A solution of **15** (500 mg, 1.20 mmol, 1.0 eq.) in anhydrous pyridine (50 mL) was cooled to 0°C, then TsCl (274.5 mg, 1.44 mmol, 1.2 eq.) and DMAP (14.7 mg, 0.12 mmol, 0.1 eq.) was added. The reaction mixture was stirred at room temperature overnight under a nitrogen atmosphere. The solvent was removed in vacuum, and the residue was purified by silica gel flash chromatography with DCM:MeOH gradually from 20:1 to 15:1 to give **16** as a white solid (411 mg, 0.71 mmol, 60%). **TLC** (Rf=0.6, DCM: MeOH =9:1, stained by 10% H_2SO_4 in MeOH). **^{1}H NMR:** (400 MHz, MeOD) δ 7.82 (d, *J* = 8.3 Hz, 2H), 7.46 (d, *J* = 8.0 Hz, 2H), 4.28 (dd, *J* = 9.9, 2.2 Hz, 1H), 4.11 – 4.01 (m, 2H), 3.99 (dd, *J* = 10.5, 1.4 Hz, 1H), 3.90 – 3.83 (m, 1H), 3.81 – 3.75 (m, 4H), 3.59 (dd, *J* = 9.2, 1.4 Hz, 1H), 3.33 (dt, *J* = 3.2, 1.6 Hz, 1H), 3.33 (dt, *J* = 3.2, 1.6 Hz, 1H), 3.22 – 3.15 (m, 2H), 2.47 (s, 3H), 2.22 (dd, *J* = 12.9, 4.9 Hz, 1H), 2.19 – 2.14 (m, 3H), 1.89 (dd, *J* = 12.8, 11.5 Hz, 1H).**^{13}C NMR:** (101 MHz, MeOD) δ 175.91, 171.64, 146.39, 134.18, 131.02,

129.17, 96.65, 73.56, 71.95, 69.79, 69.18, 67.57, 54.41, 53.25, 40.85, 26.49, 21.58, 5.63. **ESI-HRMS**: Calcd. for $C_{20}H_{29}NO_{11}SSeNa$ [M+Na]$^+$ = 594.0519, found 594.0512

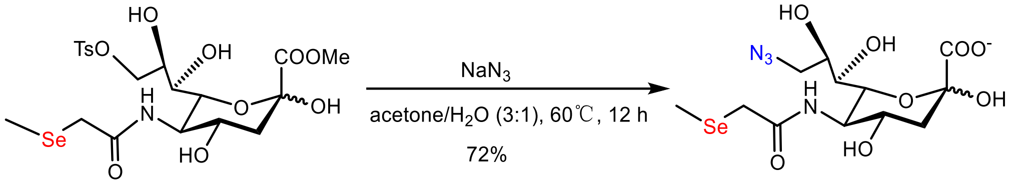

16 **17**

(*4S,5R*)-6-((*1R,2R*)-3-azido-1,2-dihydroxypropyl)-2,4-dihydroxy-5-(2-(methylselanyl)acetamido)tetrahydro-*2H*-pyran-2-carboxylate (17): 16 (400 mg, 0.70 mmol, 1.0 eq.) was dissolved in acetone (15 mL) and H_2O (5 mL), and then NaN_3 (227.5 mg, 3.5 eq.) was added. The reaction was stirred overnight at reflux at 60°C. After removal of the solvent in vacuum, the residue was purified by silica flash chromatography with DCM: MeOH gradually from 4:1 to 1:1. Further purification via normal phase HPLC (XBridge Prep amide column,19 mm×250 mm, 5 µm) gave **17** as a light yellow solid (215 mg, 0.50 mmol, 72%), eluted with 0-95% water in ACN at the flow rate of 7 mL/min. **TLC** (Rf=0.4, DCM:MeOH:H_2O =3:2:0.5, stained by 10% H_2SO_4 in MeOH). **^{1}H NMR:** (400 MHz, D_2O) δ 4.09 – 3.98 (m, 2H), 3.97 – 3.85 (m, 2H), 3.71 – 3.59 (m, 2H), 3.49 (dd, *J* = 13.2, 5.6 Hz, 1H), 3.30 – 3.21 (m, 2H), 2.28 – 2.20 (m, 1H), 2.19 – 2.10 (m, 3H), 1.84 (dd, *J* = 12.8, 11.6 Hz, 1H). **^{13}C NMR:** (101 MHz, D_2O) δ 176.67, 174.81, 96.39, 70.09, 69.04, 67.09, 53.89, 52.48, 39.56, 25.84, 4.98. **ESI-HRMS**: Calcd. for $C_{12}H_{19}N_4O_8Se^-$ [M-H]$^-$ = 427.0373, found 427.0368

Supplementary Note 2: CyTOF gating strategy

All samples were gated using same gating strategy and analyzed by FlowJo software. Specifically, prior to loading onto the CyTOF instrument, samples were mixed with element calibration beads exhibiting positive Eu signal to standardize the mass cytometry data and account for signal variability. When gating cells, ^{151}Eu/^{153}Eu was used initially to eliminate beads with high Eu signal and select the cell group. Subsequently, ^{191}Ir/^{193}Ir was employed to identify DNA-positive single cells. Following DNA-positive selection, cisplatin (^{195}Pt) negative events were gated as live cells. Cisplatin is a stain that covalently binds to cellular proteins, preferentially labeling cells with compromised cell membranes. Take the samples of vehicle- and Ac_4ManNSe-treated HeLa cells for examples, the gating strategy is as follows:

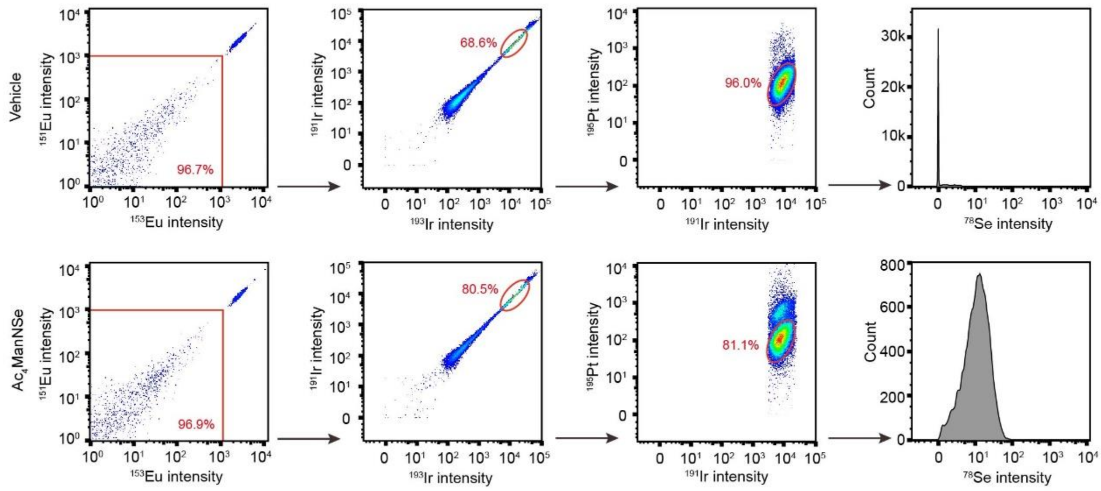

Supplementary Note 3: Flow cytometry gating strategy

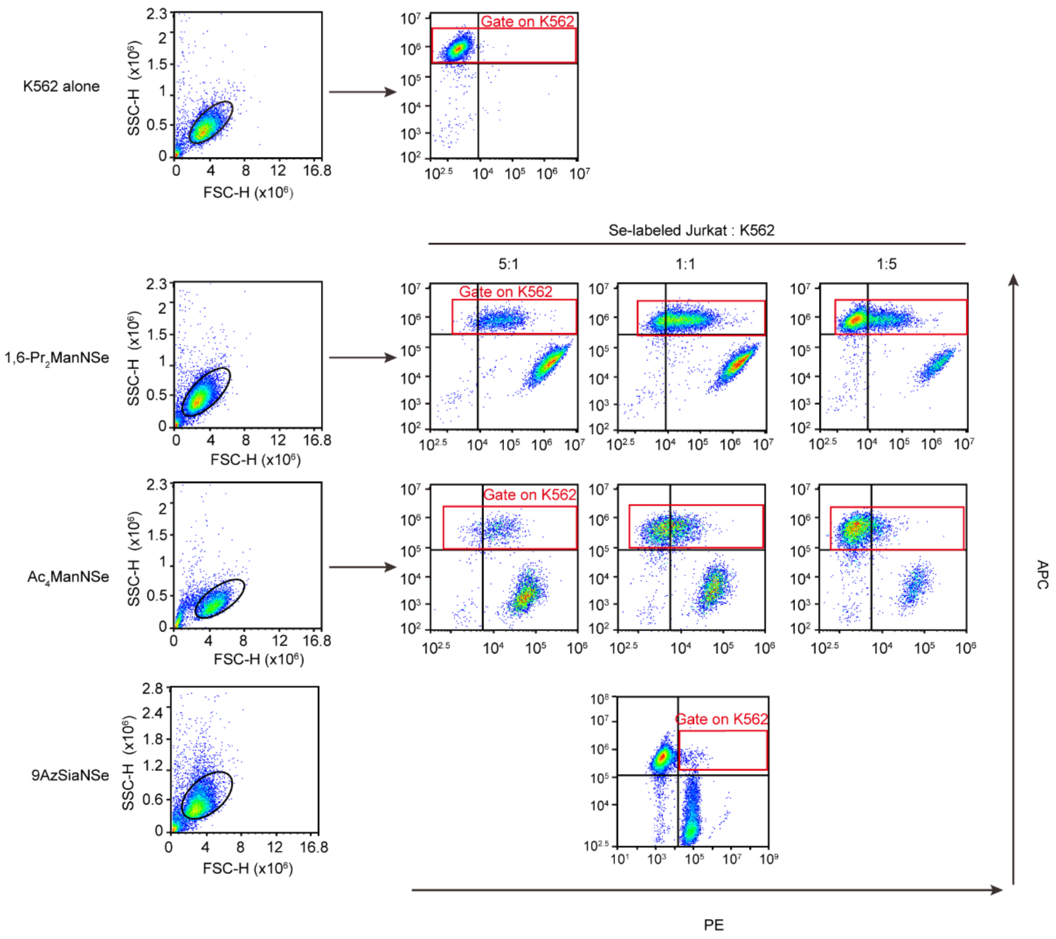

Supplementary Note 4: Calculation of the number of selenoproteins in a human body

Normally, there are around 15 mg of selenium and 50 trillion cells in an adult body (70 kg), and around 80% of selenium is found in selenoproteins. The molecular weight of selenium is 78.96 g/mol. Avogadro's constant is 6.02×10^{23}. According to the above information, the number of selenoproteins per cell in a human body can be extrapolated and calculated as follows:

15 mg/1000/78.96 $\times (6.02 \times 10^{23}) \times 80\%/(5 \times 10^{13}$ cells$) = 1.8 \times 10^{6}$ selenoprotein per cell

Supplementary Table 1: The instrumental and operational parameters of ICP-MS solution analysis

ICP-MS	Parameter
Forward power (W)	1600
Nebulizer gas (L min^{-1})	1
Cool gas (L min^{-1})	18
Auxiliary gas (L min^{-1})	1.2
Spray chamber	Cyclone
Interface	Ni Cone
Nebulizer	Concentric
Isotope	^{78}Se
Uptake Rate (mL min^{-1})	0.3

Supplementary Table 2: Instrumental parameters of LA-ICP-MS for selenoprotein analysis on PVDF membrane

	Parameter
**ICP-MS**	
RF power	1600W
Nebulizer gas	1.2 L/min
Auxiliary gas	1.2 L/min
Plasma gas	18.0 L/min
Measurement mode	Standard mode
Cone materials	Ni
Dwell time	10 ms
Isotope	^{78}Se
**Laser ablation system**	
Laser wavelength	193 nm
Sample introduction system	ARIS
Ablation mode	Linear ablation
Spot size	100 µm
Laser fluency	0.90 J/cm^2
Laser repetition rate	100 Hz
Line scan velocity	1 mm/s
He flow of inner cell	0.3 L/min
He flow of outer cell	0.3 L/min

Supplementary Table 3: Instrumental parameters of LA-ICP-TOF MS for mouse tissue sialoglycan imaging

	Parameter
**ICP-TOFMS**	
RF power	1550W
Nebulizer gas	0.89 L/min
Auxiliary gas	0.8 L/min
Plasma gas	14.0 L/min
Measurement mode	CCT mode
CCT flow	4.5 mL/min
m/z range	14-256
Cone materials	Ni
Notch mass	28, 32, 40, 80
Injector diameter	2 mm
TOF extraction time	46 µs
Waveform	516
**Laser ablation system**	
Laser wavelength	193 nm
Sample introduction system	ARIS
Ablation mode	Spot ablation
Spot size	20 µm
Laser fluency	0.90 J /cm^2
Stage movement	5000 µm/s
He flow of inner cell	0.45 L/min
He flow of outer cell	0.15 L/min
Laser repetition rate	250 Hz

Supplementary Table 4: Instrumental parameters of LA-ICP-MS for mouse tissue sialoglycan quantification *in situ*

	**Parameter**
**ICP-MS**	
RF power	1600W
Nebulizer gas	1.2 L/min
Auxiliary gas	1.2 L/min
Plasma gas	18.0 L/min
Measurement mode	Standard mode
Cone materials	Ni
Dwell time	10 ms
Isotope	^{78}Se
**Laser ablation system**	
Laser wavelength	193 nm
Sample introduction system	ARIS
Ablation mode	Spot ablation
Spot size	20 μm
Offset of Scans	40 μm
Laser fluency	0.90 J /cm^2
Stage movement	2000 μm/s
He flow of inner cell	0.3 L/min
He flow of outer cell	0.3 L/min
Laser repetition rate	100 Hz

Supplementary Table 5: RT-qPCR primers

Gene	Primer sequence (5'-3')
Human-*GAPDH*	Forward CCATGTTCGTCATGGGTGTGAACCA<br>Reverse GCCAGTAGAGGCAGGGATGATGTTC
Human-*GPX1*	Forward TCGGTGTATGCCTTCTCGG<br>Reverse CGTTCTCCTGATGCCCAAA
Human-*GPX4*	Forward AGGGAGTAACGAAGAGATCA<br>Reverse GATGAGGAACTTGGTGAAGT
Human-*SelK*	Forward CGGACAAGTGTTGGACAGC<br>Reverse GCCACGCAGATGATTGATT
Human-*SelR*	Forward CGTTCACCGAGACCATTC<br>Reverse GCCTTTAGGGACAAACTTCA
