# Supplementary material for: Selenium-based metabolic oligosaccharide engineering strategy for quantitative glycan detection
Source: Nat Commun. 2023 Dec 13;14:8281. doi: 10.1038/s41467-023-44118-w (PMC10719347; doi:10.1038/s41467-023-44118-w)
Supplement: Supplementary file 3 — Description of Additional Supplementary Files [file 41467_2023_44118_MOESM3_ESM.pdf]

Description of Additional Supplementary Files

File Name: Supplementary Data 1
Description: Peptide spectrum matches in the pGlyco3 searches

File Name: Supplementary Data 2
Description: Data 2 MS1 matches in the SESTAR++ searches

File Name: Supplementary Data 3
Description: Intact N-glycopeptides identified by SeMOE and pGlyco3
