# Supplementary material for: Selenium-based metabolic oligosaccharide engineering strategy for quantitative glycan detection
Source: Nat Commun. 2023 Dec 13;14:8281. doi: 10.1038/s41467-023-44118-w (PMC10719347; doi:10.1038/s41467-023-44118-w)
Supplement: Supplementary file 7 — Reporting Summary [file 41467_2023_44118_MOESM7_ESM.pdf]

nature portfolio

Corresponding author(s): Ran Xie, Meng Wang

Last updated by author(s): Nov 21, 2023

Reporting Summary

Nature Portfolio wishes to improve the reproducibility of the work that we publish. This form provides structure for consistency and transparency in reporting. For further information on Nature Portfolio policies, see our Editorial Policies and the Editorial Policy Checklist.

Statistics

For all statistical analyses, confirm that the following items are present in the figure legend, table legend, main text, or Methods section.

n/a	Confirmed	
☐	☒	The exact sample size (*n*) for each experimental group/condition, given as a discrete number and unit of measurement
☐	☒	A statement on whether measurements were taken from distinct samples or whether the same sample was measured repeatedly
☐	☒	The statistical test(s) used AND whether they are one- or two-sided<br>*Only common tests should be described solely by name; describe more complex techniques in the Methods section.*
☒	☐	A description of all covariates tested
☒	☐	A description of any assumptions or corrections, such as tests of normality and adjustment for multiple comparisons
☐	☒	A full description of the statistical parameters including central tendency (e.g. means) or other basic estimates (e.g. regression coefficient) AND variation (e.g. standard deviation) or associated estimates of uncertainty (e.g. confidence intervals)
☐	☒	For null hypothesis testing, the test statistic (e.g. *F*, *t*, *r*) with confidence intervals, effect sizes, degrees of freedom and *P* value noted<br>*Give P values as exact values whenever suitable.*
☒	☐	For Bayesian analysis, information on the choice of priors and Markov chain Monte Carlo settings
☒	☐	For hierarchical and complex designs, identification of the appropriate level for tests and full reporting of outcomes
☒	☐	Estimates of effect sizes (e.g. Cohen's *d*, Pearson's *r*), indicating how they were calculated

Our web collection on statistics for biologists contains articles on many of the points above.

Software and code

Policy information about availability of computer code

Data collection	Cell fluorescence microscopy imaging was performed on a Zeiss LSM 700 laser scanning confocal system. Flow cytometry analysis was performed on a BD LSRFortessa Flow Cytometer system or an ACEA NovoCyte benchtop flow cytometer. Flow cytometry sorting was conducted on a BD FACSAria™ III Sorter. In-gel fluorescence scanning was conducted from a Typhoon FLA 9500 (GE Healthcare). Images of Coomassie Brilliant Blue-stained gels were collected on a ChemiDoc XRS+ (Bio-Rad). DMB analysis data were collected on an Aligent 1260 RP-HPLC system. CyTOF data were recorded on a CyTOF-Helios (Fluidigm). ICP-MS data for solution samples were performed by a solution nebulization ICP-MS (PerkinElmer, NexION 300D, USA). LA-ICP-MS imaging of tissues was performed by a LA-ICP-TOFMS (TOFWERK AG, Thun, Switzerland) with HDIP software (Teledyne Photon Machines, Bozeman, USA), and laser image viewer software (TOFWERK AG, Thun, Switzerland). UDP-GalNSe level data were obtained from UPLC-TSQ-MS/MS (Thermo Fisher Scientific).N-glycoproteomics data were obtained from an Orbitrap Fusion Lumos Tribrid Mass Spectrometer with an EASY-Spray ionization source (Thermo Fisher Scientific). RT-qPCR was conducted on a StepOnePlus™ real-time PCR System (Thermo Fisher Scientific). NMR spectra were recorded on a Bruker-400 MHz NMR and a Bruker-500 MHz NMR instrument.
Data analysis	ZEN lite/ImageJ v3.0 was used for image analysis. NovoExpress and Flowjo v10 were used for flow cytometry data and CyTOF data analyzing. Graphical data were plotted and statistical analysis was performed using Graphpad Prism v8.0.2 (263). DMB data analysis was conducted on Origin Lab 2022. Microsoft Office Excel 2020, Origin Lab 2020, and Iolite Software v3.6 on Igor Pro 7 (WaveMetrics, USA) were used for LA-ICP-MS imaging analysis. The Orbitrap mass raw data were analyzed using pGlyco3 and SESTAR++. MestReNova v12.0.3. was used for NMR spectrum analysis. StepOne™ Software v2.3. was used for RT-qPCR data analysis. Chemdraw Professional v21.0.0 was used to draw chemical structures. Adobe illustrator CS6 was used for graph assembling.

For manuscripts utilizing custom algorithms or software that are central to the research but not yet described in published literature, software must be made available to editors and reviewers. We strongly encourage code deposition in a community repository (e.g. GitHub). See the Nature Portfolio guidelines for submitting code & software for further information.

Data

Policy information about availability of data

All manuscripts must include a data availability statement. This statement should provide the following information, where applicable:

- Accession codes, unique identifiers, or web links for publicly available datasets
- A description of any restrictions on data availability
- For clinical datasets or third party data, please ensure that the statement adheres to our policy

The mass spectrometry data, as well as all spectra for identified glycopeptides from different samples, have been deposited to the ProteomeXchange Consortium (http://proteomecentral.proteomexchange.org) via the PRIDE partner repository43 with the dataset identifier PXD042137 [http://www.ebi.ac.uk/pride/archive/projects/PXD042137] (N-glycoproteomics and SESTAR++ analysis based on SeMOE). The remaining data are available within the Article, Supplementary Information or Source Data file. Source data are provided with this paper.

Research involving human participants, their data, or biological material

Policy information about studies with human participants or human data. See also policy information about sex, gender (identity/presentation), and sexual orientation and race, ethnicity and racism.

Reporting on sex and gender	N/A
Reporting on race, ethnicity, or other socially relevant groupings	N/A
Population characteristics	N/A
Recruitment	N/A
Ethics oversight	N/A

Note that full information on the approval of the study protocol must also be provided in the manuscript.

Field-specific reporting

Please select the one below that is the best fit for your research. If you are not sure, read the appropriate sections before making your selection.

☒ Life sciences ☐ Behavioural & social sciences ☐ Ecological, evolutionary & environmental sciences

For a reference copy of the document with all sections, see nature.com/documents/nr-reporting-summary-flat.pdf

Life sciences study design

All studies must disclose on these points even when the disclosure is negative.

Sample size	Sample sizes for in vivo experiments were used on the basis of ensuring results obtained were of a representable quantity. A population range of at least 3 mice per group was used to ensure statistical power. For in vitro studies, sample size of at least 3 was used in each experiment. Sample size and number of independent experiments are stated in the figure legends.
Data exclusions	No data were excluded.
Replication	Replication was successful in all cases. The number of independent experiments is provided in the figure legends.
Randomization	Mice were randomly selected before further treatment. Cells and other samples were also randomly allocated into different groups before treatment.
Blinding	For the SeMOE in vivo and LA-ICP-MS imaging, Changjiang Wang who collected the data was blind to group allocation during the treatments and data analysis. The operation of ICP-MS was performed by Lingna Zheng who was blind to the group allocation during the treatments. For the other experiments, no blinding was performed, because the acquisition and analysis methods require human intervention to make comparisons between experiments.

Reporting for specific materials, systems and methods

We require information from authors about some types of materials, experimental systems and methods used in many studies. Here, indicate whether each material, system or method listed is relevant to your study. If you are not sure if a list item applies to your research, read the appropriate section before selecting a response.

Materials & experimental systems

n/a	Involved in the study	
☐	☒	Antibodies
☐	☒	Eukaryotic cell lines
☒	☐	Palaeontology and archaeology
☐	☒	Animals and other organisms
☒	☐	Clinical data
☒	☐	Dual use research of concern
☒	☐	Plants

Methods

n/a	Involved in the study	
☒	☐	ChIP-seq
☐	☒	Flow cytometry
☒	☐	MRI-based neuroimaging

Antibodies

Antibodies used

Streptavidin-Alexa Fluor 488 (Thermo, S32354, 1:2,000), streptavidin-Alexa Fluor 555 (Thermo, S32355, 1:2,000), anti-mouse CD80-PE (Biolegend, 104707, 1:200), and anti-mouse CD206-APC (Biolegend, 141707, 1:200) , biotinylated Sambucus Nigra Lectin (SNA) (VectorLabs, B-1305-2, 2 mg/mL stock solution, 1:400) , biotinylated Maackia Amurensis Lectin II (MAL II)(VectorLabs, B-1265-1, 1 mg/mL stock solution, 1:200) for Flow cytometry analysis.

Validation

Manuscript has been validated for the application on the manufacturer's website. Below we list one reference for each antibody.
1. Streptavidin-Alexa Fluor 488 (Thermo, S32354, 1:2,000) DOI: 10.1074/jbc.M507130200
2. Streptavidin-Alexa Fluor 555 (Thermo, S32355, 1:2,000) DOI: 10.1021/nl072460x
3. Anti-mouse CD80-PE (Biolegend, 104707, 1:200) DOI: 10.1074/mcp.M112.025205
4. Anti-mouse CD206-APC (Biolegend, 141707, 1:200) DOI: 10.1038/s41596-021-00644-9
5. biotinylated Sambucus Nigra Lectin (SNA)(VectorLabs, B-1305-2, 2 mg/mL stock solution, 1:400) DOI: 10.1038/s41467-023-39119-8
6. biotinylated Maackia Amurensis Lectin II (MAL II)(VectorLabs, B-1265-1, 1 mg/mL stock solution, 1:200) DOI: 10.1038/s41467-023-39119-8

Eukaryotic cell lines

Policy information about cell lines and Sex and Gender in Research

Cell line source(s)

Cell lines HeLa (CCL-2), A549 (CRM-CCL-185), 293T (CRL-3216), Hep G2 (HB-8065), SW620 (CCL-227), 4T1 (CRL-2539), CT26 (CRL-2638), B16-F10 (CRL-6475), RAW 264.7 (TIB-71), MCF-7 (HTB-22), MCF 10A (CRL-10317), Jurkat E6-1 (TIB-152), K562 (CCL-243), RM-1 (CRL-3310), T24 (HTB-4), HMC3 (CRL-3304), MRC-5 (CCL-171), SV-HUV-1 (CRL-9520) and HK-2 (CRL-2190) were all purchased from the American Type Culture Collection (ATCC). HCCC-9810 cells (TCHu 17) were purchased from the Institute of Biochemistry and Cell Biology, Shanghai Institutes for Biological Sciences, Chinese Academy of Sciences, Shanghai, China. GCs and oocytes were isolated from ovaries of 3-4 week-old ICR female mice.

Authentication

No further authentication was performed.

Mycoplasma contamination

All cell lines were tested negative for mycoplasma contamination.

Commonly misidentified lines
(See ICLAC register)

None.

Animals and other research organisms

Policy information about studies involving animals; ARRIVE guidelines recommended for reporting animal research, and Sex and Gender in Research

Laboratory animals

Male BALB/c mice at 6-8 weeks of age and female ICR mice at 3-4 weeks of age were purchased from GemPharmatech Co. Ltd., Nanjing, China, and kept under specific pathogen-free (SPF) conditions with free access to standard food and water (at 20–24°C, relative humidity of 40–60%, a 12h light/dark cycle).

Wild animals

This study did not involve wild animals.

Reporting on sex

Wild-type (WT) BALB/c male mice were used for SeMOE in vivo, and ICR female mice were used for isolation of primary GCs and oocytes.

Field-collected samples

This study did not involve samples collected from field.

Ethics oversight

All animal experiments were approved by Nanjing University Institutional Animal Care and Use Committee.

Note that full information on the approval of the study protocol must also be provided in the manuscript.

Flow Cytometry

Plots

Confirm that:

☒ The axis labels state the marker and fluorochrome used (e.g. CD4-FITC).

☒ The axis scales are clearly visible. Include numbers along axes only for bottom left plot of group (a 'group' is an analysis of identical markers).

☒ All plots are contour plots with outliers or pseudocolor plots.

☒ A numerical value for number of cells or percentage (with statistics) is provided.

Methodology

Sample preparation	SeMOE-treated cells were trypsinized, transferred into 96-well tissue culture plates, centrifuged (400 g, 5 min, 4°C) and washed three times with 1% FBS (v/v) in cold PBS. For click reaction experiment, cells were resuspended in PBS containing 0.5% FBS (v/v), 50 μM alkyne-biotin, premixed BTTAA-CuSO4 complex (50 μM CuSO4, BTTAA/CuSO4 6:1) and 2.5 mM freshly prepared sodium ascorbate, followed by reaction at 4°C for 10 min. After three washes with 1% FBS (v/v) in cold PBS, cells were incubated with 1 μg/mL Streptavidin Alexa Fluor 488 conjugate at 4°C for 30 min, followed by three washes with 1% FBS (v/v) in cold PBS.<br><br>For sialic acid level analysis, SeMOE-treated cells were incubated with 5 μg/mL biotinylated SNA or MAL II in 1% FBS in PBS at 4 °C for 1 h. After three washes with 1% FBS (v/v) in cold PBS, cells were incubated with Streptavidin Alexa Fluor 555 conjugate (1 μg/mL) at 4 °C for 30 min, followed by three washes with 1% FBS (v/v) in cold PBS.<br><br>For Jurkat-K562 trogocytosis and flow cytometry sorting, Dil-PE and biotinylated labeled Jurkat cells, were co-cultured with Did-APC labeled K562 cells (2 million cells/mL) in complete 1640 medium, respectively. Co-incubations were set up in 6-well plates at varied cell number ratios (Jurkat:K562 at 5:1, 1:1, 1:5), with total cells at a cell number of 8 million/well. Cell mixtures were centrifuged at 150 g for 30 s to favor cell contact, and co-incubated with or without 0.2 mg/mL sialidase for 2 h at 37°C. After co-incubation, cells were centrifuged (400 g, 4 min, 4°C), washed three times with 2% FBS (v/v) in cold PBS. Then, the co-incubation of Dil-PE labeled Jurkat cell and Dil-APC labeled K562 cells were directly used for flow cytometry analysis and sorting, while the co-culture of biotinylated Jurkat cells and Dil-APC labeled K562 cells were additionally incubated with 1 μg/mL streptavidin-Alexa Fluor 555 (PE) in the dark at 4°C for 30 min. After three washes with 2% FBS (v/v) in cold PBS, cells were applied to for flow cytometry analysis and sorting.
Instrument	Flow cytometry analysis was performed on a BD LSRFortessa Flow Cytometer system or an ACEA NovoCyte benchtop flow cytometer. Flow cytometry sorting was conducted on a BD FACSAria™ III Sorter.
Software	BD FACSuite Flow Cytometry Software or NovoExpress was used for signal collection.<br>Flowjo v10 or NovoExpress was used for data analyzing.
Cell population abundance	Cell purity was more than 99% determined by purity checks for representative samples.
Gating strategy	For general flow cytometry analysis, cells were first gated using FSC/SSC characteristics, and then combining FITC/APC/PE area signals to analyze labeling efficiency. For CyTOF analysis, 151Eu/153Eu was used initially to eliminate beads with high Eu signal and select the cell group. Subsequently, 191Ir/193Ir was employed to identify DNA-positive single cells. Then cisplatin (195Pt) negative events were gated as live cells.

☒ Tick this box to confirm that a figure exemplifying the gating strategy is provided in the Supplementary Information.
